# ﻿The polyphyletic Caucasus-centred *Campanula* subg. *Scapiflorae* (Campanulaceae) revisited with a newly circumscribed *C.* sect. *Tridentatae* for its core clade

**DOI:** 10.3897/phytokeys.243.120908

**Published:** 2024-06-25

**Authors:** Nana Silakadze, Marine Mosulishvili, Thomas Borsch, Norbert Kilian

**Affiliations:** 1 Botanischer Garten und Botanisches Museum Berlin, Freie Universität Berlin, Königin-Luise-Str. 6–8, 14195 Berlin, Germany Freie Universität Berlin Berlin Germany; 2 Ilia State University, Cholokashvili Avenue 3/5, 0162 Tbilisi, Georgia Ilia State University Tbilisi Georgia

**Keywords:** Bellflower family, Caucasus range, molecular phylogenetics, taxonomy

## Abstract

*Campanula* L. is among the genera with the highest number of endemics in the Caucasus ecoregion. A group of attractive alpine and subalpine perennial rosette plants with short single-flowered stems centred in the Caucasus has been treated as Campanulasubg.Scapiflorae or at other ranks, with considerably varying circumscription and classification. Molecular phylogenetic analysis of three plastid DNA regions (*trnK/matK*, *petD*, *rpl16*) of a strongly extended sampling, comprising 23 of the 27 commonly accepted taxa (85%) with 330 accessions built on and guided by the results of our previous study of the group, confirmed the polyphyly of C.subg.Scapiflorae in any of its circumscriptions. The core clade of the group comprises exclusively endemics and near-endemics of the Caucasus and is treated here as C.sect.Tridentatae in a revised circumscription. The phylogenetic relationships of the disparate other elements of the *Scapiflorae* group are outlined.

## ﻿Introduction

With approximately 600 species (Lammers 2007; Mansion et al. 2012; Jones et al. 2017), *Campanula* in the wide sense is among the one hundred largest plant genera (Frodin 2004), representing also the largest genus in the family Campanulaceae (order Asterales). Although *Campanula* was formally established by Linnaeus, it was first described more than 200 years earlier by Fuchs (1542), based on the species later named *C.trachelium* L. *Campanula* includes mostly perennial herbs with alternate leaves, bell-shaped, mostly purplish blue, pentamerous, bee-pollinated flowers and capsule fruits (Candolle 1830; Boissier 1875; Fedorov 1957). The members of the genus are found in forests, meadows, steppes and semi-deserts in temperate and subtropical zones of the northern hemisphere and is most abundant and diverse in the subalpine and alpine zones of mountain ranges (Fedorov 1957; Kovačić 2004, Jones et al. 2017). In the Caucasian flora, *Campanula* is one of the 17 genera with the highest number of endemic species (Dolukhanov 1966; Zazanashvili and Mallon 2009).

One group of species in *Campanula*, hitherto recognized as C.subg.Scapiflorae (Boiss.) Oganesian, is mainly endemic to the Caucasus. The group is characterised by single flowers, basal leaf rosettes and preference of alpine and subalpine habitats with rocky substrate (Silakadze et al. 2019). Sharing reflexed appendages between the calyx lobes and 3-locular capsules dehiscing down to the base led Fedorov (1957) to conclude that they belong to the core of *Campanula*. This core group corresponds to the large clade CAM 17 found by Mansion et al. (2012) in their overall analysis of *Campanula* and allies, which also includes *C.latifolia* L., the type of the name *Campanula*. In contrast, the genus in any of its current wider circumscriptions is excessively paraphyletic to a number of segregate genera mostly differing in floral traits (Mansion et al. 2012; Xu and Hong 2020).

The *Scapiflorae* group is an example of a complex species group in *Campanula* in the sense that different authors arrived at very different conclusions on the numbers and boundaries of the taxa to be recognized in this group. Whereas Ruprecht (1867), Boissier (1875) and Fomin (1905) had recognized 13, eight and 12 species, respectively, Fedorov (1957) and Kharadze (1949, 1976) recognized many more species (26 and 24, respectively). Oganesian (2000) accepted a similar number of taxa (25), of which 20 were at the species level and five additional subspecies. Seven of the species accepted by Fedorov (1957) and Kharadze (1976) were treated by her as synonyms, whereas, on the other hand, she added eight more species not included to the *Scapiflorae* before. In the most recent treatment Victorov (2001, 2002) recognized 11 species with seven subspecies. Whereas Mansion et al. (2012) just placed several of these species in one of the major *Campanula* clades (CAM 17), the addition of further molecular characters by Jones et al. (2017) and Silakadze et al. (2019) revealed many of them as part of a well-supported subclade of CAM 17, whereas others appeared distantly. However, several species hitherto classified in *Scapiflorae* remained unsampled.

The majority of species that were considered as part of the *Scapiflorae* group are endemics of the Caucasus. The distribution area of two species *Campanulaledebouriana* Trautv. and *C.minsteriana* Grossh. extends from the Caucasus into Anatolia and adjacent mountains south-eastwards; two species, *C.bornmuelleri* Nábělek and *C.pulvinaris* Hausskn. & Bornm., occur in Anatolia only, and two further are European, of which one (*C.alpina* Jacq.,) is restricted to the eastern Alps and the Carpathians, the other (*C.orbelica* Pančić) to the mountains of the central and eastern Balkans (Ronikier and Zalewska-Gałosz 2014); two species, *C.capusii* (Franch.) Fed. and *C.lehmanniana* Bunge, occur in middle Asia and two species, *C.chamissonis* Fed. and *C.dasyantha* M. Bieb., are distributed in North-East Asia. Moreover, recent phylogenetic studies (Silakadze et al. 2019) revealed that *C.ciliata* Steven, the type of the name C.subg.Scapiflorae, is only distantly related to the core clade of this group, and more closely related to *C.latifolia*, the type species of the name *Campanula*.

The present paper has three aims: Based on a considerably increased sampling of *Campanula* species in the Caucasus region and species previously assigned to C.subg.Scapiflorae (1) to further evaluate the composition of the *Scapiflorae* clade (in the sense of Silakadze et al. 2019), (2) to update the classification of the *Scapiflorae* group at the supraspecific level; and (3) to further examine phylogenetic relationships within this clade.

## ﻿Materials and methods

### ﻿Taxon sampling

We densely sampled the species diversity of Campanulasubg.Scapiflorae across its entire geographical range. Samples were collected during fieldwork in Armenia, Georgia, and Russia, and additional material from various countries was examined from the herbaria of B, DAG, ERE and TBI (Appendix 1). Compared to our previous study (Silakadze et al. 2019), we added nine more taxa with multiple accessions and covering a wider geographical range of the Caucasus region. These include eight Caucasian endemics, i.e., *C.anomala* Fomin, *C.ardonensis* Rupr., *C.besenginica* Fomin, *C.doluchanovii* Kharadze, *C.fominii* Grossh., *C.kadargavanica* Amirkh. & Komzha, *C.kryophila* Rupr. *C.sosnowskyi* Kharadze and the Caucasian and East Anatolian *C.ledebouriana* (see Suppl. material 3). One accession previously identified as C.cf.aucheri A. DC. (CAM217) was excluded from our extended dataset, as we were not sure about the accuracy of the sequences. For *C.doluchanovii* and *C.meyeriana* Rupr., we were able to include sequences from type material. We also included additional accessions of two species *C.ciliata* and *C.petrophila* Rupr., to test with material from different localities if these species are indeed not part of the *Scapiflorae* clade.

Our sampling of the *Scapiflorae* group contained 23 of the 27 commonly accepted taxa (85%) with 330 (271 newly included) accessions. In addition, we increased the sampling of other Caucasian *Campanula* species, located in the CAM 17C clade (Mansion et al. 2012; Jones et al. 2017; Silakadze et al. 2019), by 54 new accessions, some species from multiple localities. Overall, our sampling of *Campanula* was increased by 325 additional sequences representing three genomic regions of the plastid DNA (*trnK/matK*, *petD* and *rpl16*), more than doubling the sampling of our previous study (Silakadze et al. 2019).

### ﻿DNA extraction, amplification, sequencing, and alignment

DNA extraction, amplification, sequencing and alignment followed Silakadze et al. (2019). For newly generated sequences from the leaf tissue of older herbarium specimens, we used a CTAB protocol with extraction of three fractions for each plant sample (Borsch et al. 2003). As DNA was often much degraded and also contained secondary metabolites, we combined fractions I, II and III of each sample and cleaned them using DNeasy PowerClean Pro Cleanup Kit. DNA size (bp) and concentration (ng/μl) were checked using Fragment Analyser (www.aati-us.com), and 10 ng/μl was considered as ideal for the polymerase chain reaction (PCR). DNA samples were diluted with purified water depending on the concentration; if the concentration was less than 10 ng/μl, the amount of DNA was duplicated in the PCR mix. DNA stocks were kept at −20 °C.

For amplification and sequencing, we mostly used the same primers as in our previous study (Silakadze et al. 2019). For *petD* we used shorter primers designed by Schäferhoff (unpublished), and, in addition, we designed new, shorter internal primers for *rpl16* and *trnK*/*matK* (see Suppl. material 4). Primer design was carried out using the *Campanula* alignment published in Silakadze et al. (2019), using the program Seqstate 1.4.1 (Müller 2005).

All pherograms were checked using PhyDE version 0.9971 (Müller et al. 2010) and manually assembled using the motif-based alignment approach for non-coding plastid DNA (Kelchner 2002; Borsch et al. 2003; Löhne and Borsch 2005). Indels were coded as binary characters using the Simple Indel Coding approach (Simmons and Ochoterena 2000) as implemented in SeqState version 1.4.1 (Müller 2005). Consensus DNA sequences were submitted to ENA (European Nucleotide Archive) (www.ebi.ac.uk/ena/), using the software tool EMBL2checklists (Gruenstaeudl and Hartmaring 2019).

### ﻿Phylogenetic analysis

We used the matrix including the same plastid DNA regions (*trnK*/*matK*, *petD* and *rpl16*) as in Silakadze et al. (2019). Phylogenetic analyses were performed using maximum parsimony (MP), maximum likelihood (ML) and Bayesian inference (BI) approaches.

Maximum parsimony (MP) analysis was done in PAUP version 4.0b10 (Swofford 2002), using the parsimony ratchet settings (Nixon 1999) conducted in PRAP version 2.0b3 (Müller 2004). As ratchet parameters, we selected 1,000 iterations, unweighting 25% of the positions randomly (weight = 2), and 100 additional random cycles. Jackknife (JK) support was derived through a single heuristic search in PRAP with 10,000 replicates using tree bisection-reconnection (TBR) branch swapping and in each replicate, 36.79% of the characters were deleted.

Maximum likelihood (ML) analyses were executed with RAxML version 8.2.12 (Stamatakis 2014) on the CIPRES Science Gateway V 3.3 (Miller et al. 2011), using the CAT approximation (Stamatakis 2006) of the GTR model of the DNA partitions and BINCAT for the binary indel partitions (Stamatakis 2014). Rapid bootstrap analyses (BS) were conducted with 1,000 iterations integrated with a thorough ML search.

For Bayesian inference, the nucleotide dataset was divided into six partitions and the likelihood scores of models of sequence evolution were calculated for each using jModelTest version 2.1.7 (Darriba et al. 2012), choosing the best-fitting model under Akaike’s Information Criterion (AIC). Three partitions represented *trnK/matK* (*trnK* 5’ intron = GTR+G, *matK* gene = TVM+G, and *trnK* 3’ intron = GTR+G), two partitions *petD* (*petB-petD* intergenic spacer = TVM+I+G and *petD* exon/intron = TVM+G), and one partition the *rpl16* intron (TVM+I+G). The indel matrices were added using the restriction site model (Ronquist and Huelsenbeck 2003).

Bayesian inference analyses was carried out in MrBayes v.3.2.7.a (Ronquist et al. 2011) on CIPRES (Miller et al. 2011), with four runs and four chains each performed for 20 million generations, sampling every 5000^th^ generation. We checked convergence of the runs into stationarity by examining the average standard deviation of split frequencies and post-burn-in effective sampling size (ESS). The first 10% of trees were discarded as burn-in; the remaining trees were used to construct a 50% majority-rule consensus tree.

Additionally, Maximum parsimony (MP) and Maximum likelihood (ML) were applied to a matrix with two further species, *Campanulakadargavanica*, and *C.pulvinaris*, for which only *petD* and *rpl16* sequences were available.

## ﻿Results

### ﻿Phylogenetic analysis

The final alignment of three combined plastid genomic regions (*trnK/matK*, *petD*, and *rpl16*) containing 536 concatenated sequences (325 newly generated) had a total length of 5,361 positions, of which *trnK/matK* had 2,854, *petD* 1,099 and *rpl16* 1408. To document intraspecific variation, multiple samples for the same taxa were maintained in the alignment. In the multiple sequence alignment of *trnK/matK*, we excluded six hotspots of uncertain homology or poly-A/T microsatellites [positions 633–738; 746–779; 785–788; 2539–2627; 2754–2763; 2769–2773]. In *petD* one hotspot was excluded with poly-A [position 3681–3684] and in *rpl16* ten hotspots were excluded, including some poly A/T microsatellites or other sequence elements [positions 3962–3969; 4066–4083; 4123–4132; 4239–4249; 4380–4390; 4417–4419; 4463–4470; 4811–4820; 4835–4846; 5219–5252]. The final concatenated plastid matrix contained 4,984 bases (2,606 bp of *trnK/matK*, 1,095 bp of *petD*, and 1,283 bp of *rpl16*, respectively). Simple Indel Coding provided further 270 binary characters (104 of *trnK/matK*, 61 of *petD*, and 105 of *rpl16*).

Bayesian inference, maximum likelihood, and maximum parsimony analyses of the concatenated plastid dataset produced largely identical topologies and revealed significant statistical support values for various nodes at PP > 0.95 and BS and JK values > 70%, and strong support values at PP > 0.99 and BS and JK values > 90%.

**Figure 1.**
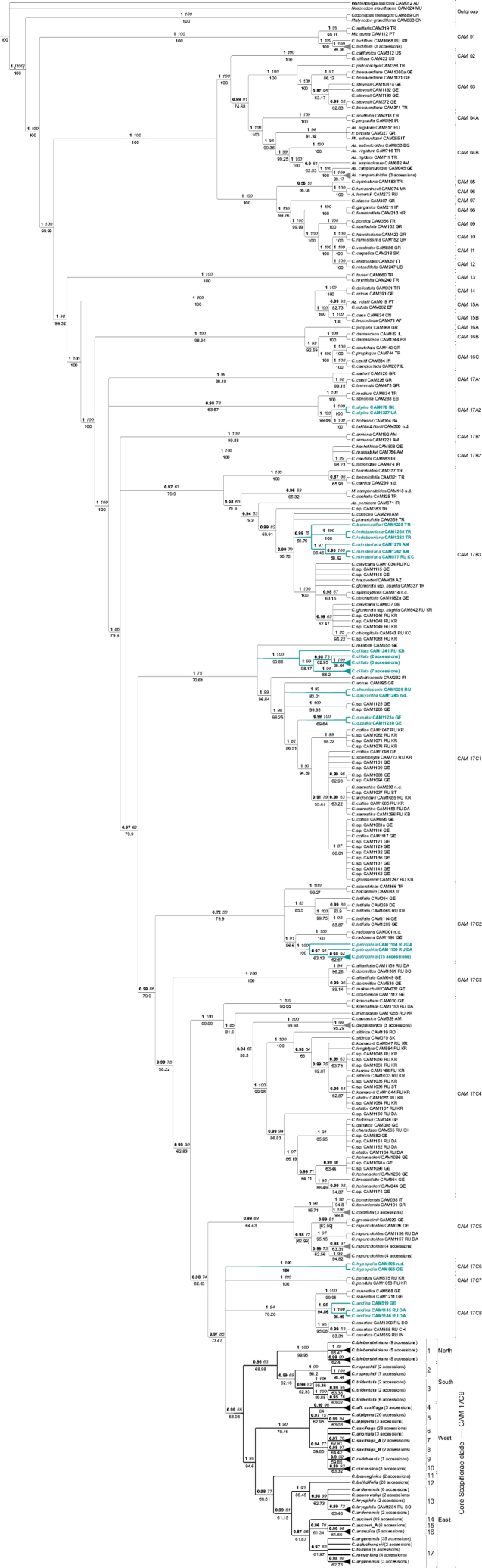
Bayesian 50% majority-rule consensus tree of the combined dataset of the Caucasian *Campanula* species based on three plastid markers (*trnK/matK*, *petD* and *rpl16*). Values above nodes indicate posterior probabilities (bold) and maximum likelihood bootstrap support (italic), values below nodes indicate maximum parsimony jackknife support, values in square brackets indicate conflicting topologies. Sample designations include the taxon name, DNA lab code, and ISO (international organization for standardization) country code, in case of the Russian part of the Caucasus also the TDWG (Biodiversity Information Standards) code of the territory (n.d. – sample not documented); for DNA lab codes and ISO codes of collapsed terminals with multiple accessions, see Suppl. materials 1, 2. Abbreviations in species names: *A.* – *Adenophora, As. – Asyneuma*, *Az. – Azorina*, *C. – Campanula*, *G. – Githopsis*, *M. – Michauxia*, *Mu. – Musschia*, *P. – Petromarula*, *Ph. – Phyteuma*. Sample designations in bold (in black) indicate species from the core *Scapiflorae* clade, sample designations in blue indicate *Scapiflorae* group members phylogenetically distant from the core clade. Subdivision of the *Scapiflorae* clade by numbered terminal clades (1-17) and geographical distribution of the lineages.

Twelve species hitherto considered as members of the *Scapiflorae* group were found distantly related to the core *Scapiflorae* clade (Fig. 1; see also Suppl. materials 1, 2, 3).

*Campanulaalpina*, which is sister to *C.hofmanii* (Pant.) Greuter & Burdet and *C.takhtadzhianii* Fed. with strong support (PP = 1, BS = 100, JK = 99.84), was resolved in clade CAM 17A2.

*Campanulabornmuelleri*, *C.ledebouriana* and *C.minsteriana* were found related to each other in a polytomy with good support (PP = 0.99, BS = 75, JK = 56.76), all nested in clade CAM 17B3.

*Campanulaciliata*, *C.dasyantha*, *C.chamissonis* and *C.dzaaku* Albov were resolved in the clade CAM 17C1 with good support (PP = 1, BS = 75, JK = 70.61). The various *C.ciliata* samples appear all in one clade which forms a trichotomy with *C.mirabilis* Albov and a clade including the remainder of CAM 17C1. In the last clade, *C.dasyantha* and *C.chamissonis* formed a sister-group with strong support (PP = 1, BS = 92, JK = 83.01), and the two *C.dzaaku* samples emerged in a separate subclade of their own with strong support (PP = 0.99, BS = 100, JK = 69.64).

*Campanulapetrophila* was found sister to *C.raddeana* Trautv. with strong support (PP = 1, BS = 91, JK = 96.6), nested in clade CAM 17C2. Notably, *C.latifolia*, the type of the genus name, fell in the same clade as *C.petrophila*.

*Campanulahypopolia* Trautv. (Clade CAM 17C6) and *C.andina* Rupr., forming a trichotomy (Clade CAM 17C8) with *C.suanetica* Rupr. and *C.ossetica* M. Bieb., were found nested in the polytomy with the *C.pendula* (Clade CAM 17C7) and the core *Scapiflorae* clade (CAM 17C9).

The additional tree (see Suppl. material 2; ML and MP), including further species of which only *petD* and *rpl16* sequences were available, revealed that also *Campanulapulvinaris* is not part of the *Scapiflorae* clade and instead nested in clade CAM 17B3 with *C.bornmuelleri*, *C.ledebouriana* and *C.minsteriana*, although without support. Notably, *C.latifolia*, the type of the genus name, fell in the same clade as *C.petrophila*, being sister in a polytomy to two clades, one containing *C.petrophila* and *C.raddeana* and another *C.sclerotricha* Boiss. and *C.trachelium*; all are nested in clade CAM 17C2, which is, however, weakly supported (PP = 0.72, BS = 50, JK = 79.9).

The majority of the taxa of the *Scapiflorae* group were resolved in clade CAM 17C9, here considered the core *Scapiflorae* clade. Within the well-supported *Scapiflorae* core clade (PP = 0.99, BS = 65, JK = 68.9; Fig. 1, see also Suppl. material 1) 17 terminal clades were resolved, which form two larger lineages and sister clades: the lineage A or north/south clade (PP = 0.86, BS = 63, JK = 68.98; Fig. 1, see also Suppl. material 1) and the strongly supported lineage B or west/east clade (PP = 1, BS = 95, JK = 94.6; Fig. 1, see also Suppl. material 1) .

The clade A1 corresponds to the *C.biebersteiniana* Roem. & Schult. accessions from the Greater Caucasus, extending to the northern part of the Caucasus region, and hence clade A1 is also referred to as north (N) clade, exhibiting strong supported (PP = 1, BS = 100, JK = 99.95).

The clade A2 contains the terminal clades 2 and 3 with the accessions of *C.ruprechtii* Boiss. and *C.tridentata* Schreb., all from the Lesser Caucasus, extending to the southern part of the Caucasus, and so this clade was also referred to as south (S) clade, likewise moderately supported (PP = 99.69, BS = 69, JK = 62.16).

The clade B1 received strong support (PP = 1, BS = 90, JK = 70.11) and comprises the terminal clades 4 through 10, including the accessions of *C.alpigena* K. Koch, *C.anomala*, *C.circassica* Fomin, *C.radchensis* Kharadze, *C.saxifraga* M. Bieb. and C.aff.saxifraga, all from the western part of the Caucasus and hence referred to as the west (W) clade.

Finally, the clade B2, which received moderate support (PP = 0.98, BS = 77, JK = 60.51), consists of the terminal clades 11 through 17 and includes the accessions of *C.ardonensis*, *C.argunensis* Rupr., *armazica* Kharadze, *C.aucheri*, *C.bellidifolia* Adams, *C.besenginica*, *C.doluchanovii*, *C.fominii*, *C.kryophila*, *C.meyeriana* and *C.sosnowskyi*, all originating from the eastern part of the Caucasus, and hence referred to as the east (E) clade.

The terminal clade 1 contains 22 accessions of *C.biebersteiniana* and shows strong support (PP = 1, BS = 100, JK = 99.95). Clade 2 covered nine accessions of *C.ruprechtii*, strongly supported (PP = 1, BS = 99, JK = 98.2). Clade 3 includes ten accessions of *C.tridentata* with good support (PP = 0.99, BS = 82, JK = 62.33; Fig. 1, see also Suppl. material 1). Clade 4 with strong support (PP = 0.99, BS = 96, JK = 64) consists of three accessions of uncertain identifications, two labelled *C.besenginica* and one *C.saxifraga*, which we all treat as C.aff.saxifraga. Clade 5 contains 23 accessions of *C.alpigena* with moderate support (PP = 0.97, BS = 75, JK = 62.95), whereas clade 6 includes three accessions of *C.anomala* and 48 of *C.saxifraga* with weak support (PP = 0.94, BS = 77, JK = 59.85). Clades 7, 8, 9 and 10 are all nested within clade 6, encompassing *C.saxifraga* A with two accessions with moderate support (PP = 0.97, BS = 70, JK = 62.91), *C.saxifraga* B also with two accessions with good support (PP = 0.98, BS = 97, JK = 64.42), *C.radchensis*, comprising seven accessions with moderate support (PP = 0.9, BS = 92, JK = 59.85), and *C.circassica* with eight accessions, also with moderate support (PP = 0.89, BS = 93, JK = 63.32; Fig. 1, see also Suppl. material 1). Clade 11 consists of two accessions of *C.besenginica*, forming a polytomy relative to all other terminal clades in this area of the tree. Clade 12 includes 20 accessions of *C.bellidifolia* and has good support (PP = 1, BS = 93, JK = 86.45), whereas clade 13, nested within clade 12, covered eight accessions of *C.ardonensis*, three of *C.kryophila* and two of *C.sosnowskyi* with good support (PP = 0.98, BS = 99, JK = 62.73). The next terminal clade 14 included 49 accessions of *C.aucheri* with moderate support (PP = 87, BS = 96, JK = 61.67), with clades 15 and 16 nested within clade 14, consisting of six accessions of *C.aucheri* A and five of *C.armazica*, with moderate or good support, respectively (PP = 0.96, BS = 70, JK = 61.34; PP = 0.99, BS = 95, JK = 61.66). Finally, clade 17, also nested within clade 14, included 38 accessions of *C.argunensis*, two of *C.doluchanovii*, six of *C.fominii* and four of *C.meyeriana*, overall with moderate support (PP = 0.97, BS = 83, JK = 61.97; Fig. 1, see also Suppl. material 1).

## ﻿Discussion

### ﻿Revised classification of the core *Scapiflorae* clade

The re-circumscription and infrageneric re-classification of the iconic bell-flower genus, *Campanula*, is still in progress (Mansion et al. 2012; Jones et al. 2017; Xu and Hong 2020). It is evident from these and further analyses that applying a phylogeny-based genus concept to *Campanula* will inevitably shatter the genus as it is known traditionally but a detailed analysis of a representative spectrum of morphological characters in a phylogenetic context will be needed to evaluate if conspicuous floral differences are not just caused by adaptive shifts as part of pollination syndromes. What applies to the genus as a whole is also true for the *Scapiflorae* group on a small scale. Silakadze et al. (2019) and the present study revealed that C.subg.Scapiflorae is an artificial assemblage but resolved a well-supported core lineage, the core *Scapiflorae* clade (Fig. 1, see also Suppl. material 1). Compared to Silakadze & al. (2019), the present study provides a much more in-depth analysis of this group, based on 330 accessions, representing 85% of the taxa of this group. The present study also fully corroborates the results of the previous study by Silakadze et al. (2019) that the core *Scapiflorae* clade and all other elements of the polyphyletic C.subg.Scapiflorae belong to the core of *Campanula* (Fig. 1, see also Suppl. material 1) defined by inclusion of *C.latifolia* as the type of the generic name in the clade CAM 17 (sensu Mansion et al. 2012 and later authors). We can thus postulate with certainty that the group will remain in *Campanula* also after a future reorganisation of the genus.

Based on research conducted by Silakadze et al. (2019), the *Scapiflorae* clade can be clearly defined and differentiated based on a combination of morphological, distributional and ecological characteristics. These include the presence of calyx appendages, leaf rosettes with a single-flowered stem, and its members typically grow in rocky habitats within the (sub)alpine zone and occur in the Caucasus region and adjacent areas to the south. One of the main results of our studies is that *Campanulaciliata*, the designated lectotype (Fedorov 1957: 256) of the name Campanulaser.Scapiflorae Boiss. and all combinations based on it, falls far outside the core *Scapiflorae* clade. Consequently, the name *Scapiflorae* is not available for a formal taxonomic recognition of the core clade. Among the authors treating the *Scapiflorae* group, only Fedorov (1957), Kharadze (1976) and Victorov (2002) formally subdivided C.subg.Scapiflorae. Fedorov (1957) and Kharadze (1976) recognized eleven and ten series, respectively, Victorov (2002) recognized seven sections and his treatment is the one most congruent with our result. Our core clade is represented in his treatment by only two species: *C.tridentata* and *C.bellidifolia*, the latter with five subspecies. They form C.sect.Tridentatae (Kharadze) Victorov, which also comprises, contrary to our results, the north-east Asian *C.dasyantha* with *C.chamissonis* as its subspecies, and the Caucasian *C.petrophila* and *C.andina*. At section rank the infrageneric epithet *Tridentatae* has priority over any other name; at series rank it is one of nine series names of equal priority for the core clade (Fedorov 1957; Kharadze 1976). Considering the phylogenetic position of the core *Scapiflorae* clade within clade CAM17, we have chosen the rank of section for the core clade, applying the name C.sect.Tridentatae in our revised circumscription restricted to the Caucasian members only. The choice of the rank, of a section, may appear a bit of a long shot given the unsettled circumscription and classification of a monophyletic genus *Campanula*. However, subgenera may likely be applied at a more inclusive level to name major clades such as CAM 1 to CAM 17, also depending if these can be recognized by morphology. For the time being it should best serve the needs of an unambiguous classification to handle the *Scapiflorae* clade at the level of a section.

Campanulasect.Tridentatae (Kharadze) Victorov ≡ Campanulaser.Tridentatae Kharadze in Zametki Sist. Geogr. Rast. 15: 25. 1949. – Type: *Campanulatridentata* Schreb.

= Campanulaser.Anomalae Fed. in Komarov (ed.), Fl. SSSR 24: 197. 1957. – Type: *Campanulaanomala* Fomin

= Campanulaser.Ardonenses Fed. in Komarov (ed.), Fl. SSSR 24: 198. 1957. – Type: *Campanulaardonensis* Rupr.

= Campanulaser.Argunenses Fed. in Komarov (ed.), Fl. SSSR 24: 192. 1957. – Type: *Campanulaargunensis* Rupr.

= Campanulaser.Aucherianae Kharadze in Zametki Sist. Geogr. Rast. 15: 25. 1949 [“*Aucheri*”]. – Type: *Campanulaaucheri* A. DC.

= Campanulaser.Bellidifoliae Fed. in Komarov (ed.), Fl. SSSR 24: 194. 1957. – Type: *Campanulabellidifolia* Adams

= Campanulaser.Besenginicae Fed. in Komarov (ed.), Fl. SSSR 24: 199. 1957. – Type: *Campanulabesenginica* Fomin

= Campanulaser.Kryophilae Kharadze in Zametki Sist. Geogr. Rast. 15: 25. 1949. – Type: *Campanulakryophila* Rupr.

= Campanulaser.Saxifragiformes Fed. in Komarov (ed.), Fl. SSSR 24: 190. 1957. – Type: *Campanulasaxifraga* M. Bieb.

In our circumscription, the section includes all taxa belonging to the core *Scapiflorae* clade. These are: *Campanulaalpigena*, *C.argunensis* (incl. *C.doluchanovii*, *C.fominii*, *C.meyeriana*), *C.armazica*, *C.aucheri*, *C.bellidifolia* (incl. *C.ardonensisC.sosnowskyi*, *C.kadargavanica*, *C.kryophila*), *C.besenginica*, *C.biebersteiniana*, *C.radchensis*, *C.ruprechtii*, *C.saxifraga* (incl. *C.anomala*, *C.circassica*) and *C.tridentata*. They are Caucasian endemics, or near endemics extending into southerly adjacent mountain ranges, growing on rocky to soil-rich substrates predominantly in the subalpine to alpine zone and are morphologically characterised by the presence of calyx appendages, leaf rosettes with short and strictly single-flowered stems. However, the combination of these characters does not represent an exclusive synapomorphy for the core *Scapiflorae* clade but has evolved independently also in the single case of *C.ciliata* (Silakadze et al. 2019), nested in the distant clade CAM 17C1 (see below).

A revised classification of the members of Campanulasect.Tridentatae will be substantiated and elaborated in two further contributions (Silakadze et al. in prep.; Silakadze and Kilian in prep.).

### ﻿Phylogenetic position of the *Scapiflorae* members excluded from Campanulasect.Tridentatae

According to our analysis, 12 species do not belong to the *Tridentatae* clade (Fig. 1, see also Suppl. material 1). These are *Campanulaalpina*, *C.andina*, *C.bornmuelleri*, *C.chamissonis*, *C.ciliata*, *C.dasyantha*, *C.dzaaku*, *C.hypopolia*, *C.minsteriana*, *C.ledebouriana*, *C.petrophila* and *C.pulvinaris.* Their phylogenetic positions are briefly discussed in the following.

The *Tridentatae* clade CAM 17C9 is part of a polytomy of exclusively Caucasian endemics, together with the three clades CAM 17C6, C7 and C8 (Fig. 1, see also Suppl. material 1). The first of these clades is represented by *Campanulahypopolia*, which was placed by Victorov in C.sect.Hypopolion (Fed.) Ogan. of his C.subg.Scapiflorae, the second is represented by *C.pendula* M. Bieb. The third includes *C.andina*, which was described from the “Andi” range of Dagestan and placed by Victorov (2002) in his C.sect.Tridentatae, together with *C.suanetica* and *C.ossetica* in an unresolved clade. Notably, none of them except the members of the *Tridentatae* clade have unbranched single flowered stems.

*Campanulaciliata* is resolved as member of clade CAM 17C1, far distant from the *Tridentatae* clade. All seven authors who have studied the *Scapiflorae* group agreed that *Campanulaciliata* belongs to that group (see Suppl. material 3) as defined by a set of mostly convergent morphological characters. The name *Scapiflorae*, with its type *C.ciliata*, thus applies to the clade CAM 17C1 (Fig. 1, see also Suppl. material 1), also containing the *Scapiflorae* members *C.chamissonis*, *C.dasyantha* and *C.dzaaku*, and the other Caucasian species *C.annae* Kolak., *C.collina* Sims, *C.grossheimii* Kharadze, *C.mirabilis*, *C.odontosepala* Boiss., *C.sarmatica* Ker Gawl., *C.sclerophylla* (Kolak.) Ogan. and *C.woronowii* Kharadze. At subgenus rank, C.subg.Annae (Kolak.) Ogan. is also available but does not have priority over *Scapiflorae*, whereas at sectional rank, C.sect.Annae would have priority for clade CAM 17C1. Within this clade, multiple accessions of the Caucasian *C.ciliata* form a trichotomy with *C.mirabilis* and the remainder of the clade, which also includes, besides the Caucasian members, *C.odontosepala* from Iran and the north-east Asian *C.dasyantha* and *C.chamissonis.* These *Scapiflorae* species and the closely related and sympatric *C.aldanensis* Fed. & Karav. were treated by Fedorov (1957) and Victorov (2002) as C.ser.Dasyanthae Fed. However, Victorov recognised only *C.dasyantha*, in which he included *C.chamissonis* as a subspecies (C.dasyanthasubsp.chamissonis) and sank *C.aldanensis* in the synonymy of the typical subspecies, a treatment we fully agree with. The placement of *C.dzaaku*, a species without calyx appendages and unusual coriaceous leaves with cartilagineously denticulate margin, was disputed. Kharadze (1949) and Fedorov (1957) placed it close to *C.ciliata*, Victorov (2002) placed it distant to the latter but as a member of *Scapiflorae*, and Oganesian (2000) did not at all include it in the *Scapiflorae*. The lack of calyx appendages was identified by Silakadze & al. (2019: fig. 4) as a synapomorphy of a subclade including *C.dzaaku* together with *C.collina*, *C.sclerophylla*, *C.woronowii* and, with a reversal in this state, *C.sarmatica*. As it turns out, clade CAM 17C1 is morphologically heterogenous and its internal relationships and character evolution require further study.

The Central to S European *Campanulaalpina*, still included by Oganesian (2000) and Victorov (2002) in the *Scapiflorae* group (see Suppl. material 3), is resolved very distantly to the *Tridentatae* clade, in the CAM 17A2 clade, sister to *C.hofmanii* and *C.takhtadzhianii*, and these in turn sister to *C.medium* L. and *C.speciosa* Pourr. (Fig. 1, see also Suppl. material 1). The morphological similarity of *C.alpina* to the *Tridentatae* members is only by convergent evolution to similar habitats and its inflorescence can, moreover, also be few-flowered (Silakadze et al. 2019). Another species closely related to *C.alpina* but not included in our study is the Balkan endemic *C.orbelica* (Ronikier and Zalewska-Gałosz 2014).

The former *Scapiflorae* species *Campanulabornmuelleri*, *C.ledebouriana*, *C.minsteriana* and *C.pulvinaris* are nested in CAM 17B3 is (Fig. 1, see also Suppl. material 1; for the *C.pulvinaris* see Suppl. material 2). They are distributed mainly in Anatolia (Turkey) and the Armenian Highlands. *C.karakuschensis* was included in the *Scapiflorae* group only by Victorov (2002) (see Suppl. material 3) and as a synonym of *C.minsteriana*, in line with Oganesian (2000), who classified that species under C.subg.Theodorovia (Kolak.) Ogan. *C.minsteriana* according to our phylogeny, is closely related to *C.ledebouriana*, corroborating the view of Fedorov (1957) and Kharadze (1976), who placed *C.minsteriana* with *C.ledebouriana* into C.ser.Ledebourianae Fed. of the *Scapiflorae* group. Also, Oganesian (2000) recognized *C.ledebouriana* as a member of the *Scapiflorae*, and additionally included *C.bornmuelleri* and *C.pulvinaris*. Our phylogenetic results confirm these to belong to one lineage together with *C.coriacea* P. H. Davis, *C.ptarmicifolia* Lam. and the members of the clade of *C.glomerata* L. (Fig. 1, see also Suppl. materials 1, 2). For unknown reasons, Fedorov (1957) and Kharadze (1976) treated *C.karakuschensis* not as conspecific with *C.minsteriana* but as a quite different species and outside the *Scapiflorae* group in C.ser.Saxicolae (Boiss.) Kharadze, together with *C.lehmanniana* and *C.capusii*. These two species are distributed in Kirgizistan and Tadzhikistan and were considered only by Victorov to belong to C.subg.Scapiflorae. They were not included in our phylogenetic analyses but with their branched inflorescences and Middle Asian distribution we postulate that they are certainly not closely related to the *Tridentatae* lineage.

The Caucasian endemic *Campanulapetrophila* is nested in clade CAM 17C2 sister to *C.raddeana*, another Caucasian endemic species, and together with *C.latifolia*, the type of the name *Campanula*, *C.trachelium* and *C.sclerotricha* (Fig. 1, see also Suppl. material 1). *C.petrophila* and *C.raddeana* have similar ecological patterns like growing only in vertical rocks, but somewhat differ in leaf shape and develop slightly branched inflorescence.

*Campanulaczerepanovii* Fed. is a little known local endemic of Dagestan with branched inflorescences and was included in the *Scapiflorae* group only by Kharadze (1949). It has not been included in any phylogenetic study but is certainly no member of C.sect.Tridentatae, because of its different stem morphology.

## ﻿Additional information

### Author ORCIDs

Marine Mosulishvili https://orcid.org/0000-0002-7181-6256

Norbert Kilian https://orcid.org/0000-0003-0890-4373

### Data availability

All of the data that support the findings of this study are available in the main text or Supplementary Information.

## Appendix

Sample list. Taxon sampling, voucher data and INSDC (International Nucleotide Sequence Database Collaboration, including GenBank/EMBL/DDBJ) accession numbers. The data are arranged in the following order: taxon name in italics in alphabetical order; unique sample identifier as used in the trees and, in square brackets where applicable, unit ID of preserved DNA sample in the GGBN data portal (Droege et al. 2014); abbreviated voucher data (country, locality, collecting date, collectors and collecting number, specimen barcode with stable URI to the digitised specimen, if available, or herbarium code and accession number); INSDC accession numbers in the following order: *trnK/matK*, *petD*, *rpl16*.

*Asyneumacampanuloides* (M. Bieb. ex Sims) Bornm.: CAM1126 [DB 41545]: Georgia, N Caucasus, Racha-Lechkhumi and Kvemo Svaneti (Lechkhumi), Tsageri district, Askhi Mountain, 1990 m, 42.55675°N, 42.62668°E, 23 July 2016, *N. Silakadze & al. 51* (B 10 1052266), PP004711, PP004386, PP004060; CAM1128 [DB 41564]: Georgia, N Caucasus, Samegrelo-Zemo Svaneti (Samegrelo), Martvili district, Taleri village, Chegola Mountain, 2430 m, 42.42293°N, 42.77655°E, 26 July 2016, *N. Silakadze & al. 53*(B 10 1052274), PP004719, PP004394, PP004068.

*Campanulaalpigena* K. Koch: CAM1144b [DB 41648]: Georgia, S Caucasus, On the border of Imereti and Samckhe-Javakheti, Adigeni district, Abastumani, Meskheti Range, Zekari Pass, 2200 m, 42.8731°N, 41.82823°E, 13 August 2016, *N. Silakadze 69b* (B 10 1052332), PP004759, PP004434, PP004108; CAM1144c [DB 41649]: *N. Silakadze 69c* (B 10 1052333), PP004760, PP004435, PP004109, from the same population as *69b*; CAM1144d [DB 41650]: *N. Silakadze 69c* (B 10 1052334), PP004761, PP004436, PP004110, from the same population as *69b*; CAM1144e [DB 41651]: Georgia, S Caucasus, On the border of Imereti and Samckhe-Javakheti, Adigeni district, Abastumani, Meskheti Range, Zekari Pass, 2160 m, 42.88198°N, 41.82453°E, 13 August 2016, *N. Silakadze 69e* (B 10 1052335), PP004762, PP004437, PP004111; CAM1144f [DB 41652]: *N. Silakadze 69f* (B 10 1052336), PP004763, PP004438, PP004112, from the same population as *69e*; CAM1144g [DB 41653]: *N. Silakadze 69g* (B 10 1052337), PP004764, PP004439, PP004113, from the same population as *69e*; CAM1144h [DB 41654]: Georgia, S Caucasus, On the border of Imereti and Samckhe-Javakheti, Adigeni district, Abastumani, Meskheti Range, Zekari Pass, 2170 m, 42.86617°N, 41.82565°E, 13 August 2016, *N. Silakadze 69h* (B 10 1052338), PP004765, PP004440, PP004114; CAM1144i [DB 41655]: *N. Silakadze 69i* (B 10 1052339), PP004766, PP004441, PP004115, from the same population as *69h*; CAM1144k [DB 41657]: *N. Silakadze 69k* (B 10 1052341), PP004767, PP004442, PP004116, from the same population as *69h*; CAM1144l [DB 41658]: *N. Silakadze 69l* (B 10 1052342), PP004768, PP004443, PP004117, from the same population as *69h*; CAM1144m [DB 41659]: *N. Silakadze 69m* (B 10 1052343), PP004769, PP004444, PP004118, from the same population as *69h*; CAM1144o [DB 41661]: *N. Silakadze 69o* (B 10 1052345), PP004770, PP004445, PP004119, from the same population as *69h*; CAM1195b [DB 41746]: Georgia, S Caucasus, On the border of Imereti and Samckhe-Javakheti, Adigeni district, Abastumani, Meskheti Range, Zekari Pass, 2250 m, 42.85592°N, 41.82845°E, 17 June 2017, *N. Silakadze & K. E. Jones 96b* (B 10 1052408), PP004825, PP004501, PP004175; CAM1217b [DB 41806]: Georgia, S Caucasus, Adjara, Shuakhevi district, Gogadzeebi village, Tbeti Mountain, 2260 m, 42.18222°N, 41.53198°E, 23 July 2017, *N. Silakadze & al. 118b* (B 10 1052468), PP004872, PP004548, PP004222; CAM1217c [DB 41807]: *N. Silakadze & al. 118c* (B 10 1052469), PP004873, PP004549, PP004223, from the same population as *118b*; CAM1217d [DB 41808]: Georgia, S Caucasus, Adjara, Shuakhevi district, Gogadzeebi village, Tbeti Mountain, 2240 m, 42.1841°N, 41.53018°E, 23 July 2017, *N. Silakadze & al. 118d* (B 10 1052470), PP004874, PP004550, PP004224; CAM1218a [DB 41809]: Georgia, S Caucasus, Guria, Chokhatauri district, daba Bakhmaro, Sakornia Mountain, 2100 m, 42.31428°N, 41.84426°E, 24 July 2017, *N. Silakadze & al. 119a* (B 10 1052471), PP004875, PP004551, PP004225; CAM1218c [DB 41811]: Georgia, S Caucasus, Guria, Chokhatauri district, daba Bakhmaro, Sakornia Mountain, 2130 m, 42.31405°N, 41.84333°E, 24 July 2017, *N. Silakadze & al. 119c* (B 10 1052473), PP004876, PP004552, PP004226; CAM1219 [DB 41812]: Georgia, S Caucasus, Adjara, Tivnari, Gomi Mountaun, 1920 m, 42.10991°N, 41.56324°E, 24 June 2017, *Z. Asanidze 120* (B 10 1052474), PP004905, PP004553, PP004227; CAM1235 [DB 41831]: Turkey, S Caucasus, Province Coruh (Artvin), Tiryal dag, above Murgul, 2150 m, 23 June 1957, *Davis & Hedge D29908* (ERE 56071), PP004910, PP004563, PP004237.

*Campanulaandina* Rupr.: CAM1146 [DB 41663]: Russian Federation, Dagestan, N Caucasus, Botlikhskiy raion, slopes above the road at the border to the Tsumadinskiy rayon, 860 m, 46.14317°N, 42.62853°E, 9 August 2016, *R. Murtazaliyev* s.n. (herb. Murtazaliyev), PP004771, PP004446, PP004120.

*Campanulaanomala* Fomin: CAM1271 [DB 41864]: Russian Federation, N Caucasus, Karachay-Cherkessia, Klukhor pass, 26 August 1954, *L. Khintibidze* s.n. (TBI 1037305), PP004923, PP004592, PP004302; CAM1286 [DB 41879]: Georgia, N Caucasus, Racha-Lechkhumi and Kvemo Svaneti (Racha), Shoda Mountain, 2300–2500 m, 20 August 1965, *R. Gagnidze & I. Mikeladze* s.n. (TBI 1037304), PP004931, PP004604, PP004278; CAM1289 [DB 41882]: Russian Federation, N Caucasus, Karachay-Cherkessia, Klukhor pass, 26 August 1954, *L. Khintibidze* s.n. (TBI 1037303), PP004933, PP004606, PP004280.

*Campanulaardonensis* Rupr.: CAM1177g [DB 41710]: Georgia, N Caucasus, Mtskheta-Mtianeti (Kazbegi), daba Stepantsminda, Dariali gorge, 1310 m, 44.62899°N, 42.73897°E, 7 June 2017, *N. Silakadze & K. E. Jones 78g* (B 10 1052372), PP004798, PP004473, PP004147; CAM1228 [DB 41825]: Russian Federation, N Caucasus, North Ossetia-Alania, River Ardon, Buron, 9 June 1985, *Yu. L. Menitsky* s.n. (ERE 69614), PP004906, PP004558, PP004232; CAM1280 [DB 41873]: Russian Federation, N Caucasus, North Ossetia-Alania, Bass. R. Ardon, near the glacier Tsey, 9 August 1954, *K. Qimeridze & D. Ochiauri* s.n. (TBI 1037311), PP004928, PP004600, PP004273; CAM1287 [DB 41880]: Russian Federation, N Caucasus, North Ossetia-Alania, Ardon, 4000 ?, 17 July 1902, *I.J. Akinfiev* s.n. (TBI 1037309), PP004932, PP004605, PP004279; CAM1299 [DB 40126]: Russian Federation, N Caucasus, North Ossetia-Alania, Fiagdon valley, 1135 m, 44.32638°N, 42.88166°E, 22 July 2019, *G. Parolly & al. 15795* (B 10 1118223), PP004942, PP004616, PP004290; CAM1302 [DB 40138]: Russian Federation, N Caucasus, North Ossetia-Alania, Sadon valley, just above the first tunnel, 1090 m, 44.02444°N, 42.84222°E, 23 July 2019, *G. Parolly & al. 15815* (B 10 1118229), PP004945, PP004619, PP004293; CAM1304 [DB 40143]: Russian Federation, N Caucasus, North Ossetia-Alania, Arkhon-Don valley, 1090 m, 44.10333°N, 42.84277°E, 23 July 2019, *G. Parolly & al. 15818* (B 10 1118227), PP004946, PP004620, PP004294.

*Campanulaargunensis* Rupr.: CAM1089k [DB 41396]: Georgia, N Caucasus, Kakheti (Tusheti), Akhmeta district, Dano village, 2130 m, 45.56724°N, 42.45041°E, 7 July 2016, *N. Silakadze & al. 12k* (B 10 1052213), PP004667, PP004342, PP004016; CAM1092 [DB 41414]: Georgia, N Caucasus, Kakheti (Tusheti), Akhmeta district, between villages Bochorna and Dochu, 2370 m, 45.57093°N, 42.40428°E, 8 July 2016, *N. Silakadze & al. 16* (B 10 1052217), PP004671, PP004346, PP004020; CAM1100 [DB 41433]: Georgia, N Caucasus, Kakheti (Tusheti), Akhmeta district, Dartlo village, 1910 m, 45.58929°N, 42.4276°E, 9 July 2016, *N. Silakadze & al. 25* (B 10 1052228), PP004681, PP004356, PP004030; CAM1104p [DB 41471]: Georgia, N Caucasus, Kakheti (Tusheti), Akhmeta district, road to Chesho village, 1940 m, 45.54396°N, 42.47186°E, 9 July 2016, *N. Silakadze & al. 29p* (B 10 1052233), PP004686, PP004361, PP004035; CAM1108a [DB 41478]: Georgia, N Caucasus, Kakheti (Tusheti), Akhmeta district, Abano Pass, 2290 m, 45.52758°N, 42.29441°E, 10 July 2016, *N. Silakadze & al. 33a* (B 10 1052237), PP004688, PP004363, PP004037; CAM1108b [DB 41479]: *N. Silakadze & al. 33b* (B 10 1052238), PP004689, PP004364, PP004038, from the same population as *33a*; CAM1110p [DB 41496]: Georgia, N Caucasus, Kakheti (Tusheti), Akhmeta district, Abano Pass, 2140 m, 45.50213°N, 42.25535°E, 10 July 2016, *N. Silakadze & al. 35p* (B 10 1052240), PP004691, PP004366, PP004040; CAM1148 [DB 41665]: Russian Federation, Dagestan, N Caucasus, Charodinsky raion, surroundings of village Gochob, in river gorge, eastern slope, 2050 m, 46.64914°N, 42.23283°E, 19 August 2016, *R. Murtazaliyev* s.n. (herb. Murtazaliyev), PP004772, PP004447, PP004121; CAM1149 [DB 41666]: Russian Federation, Dagestan, N Caucasus, Rutulskiy raion, Vug river gorge, below village Djinykh, northern rocky slope, 1810 m, 47.05497°N, 41.66447°E, 26 July 2016, *R. Murtazaliyev* s.n. (herb. Murtazaliyev), PP004773, PP004448, PP004122; CAM1169a [DB 41684]: Georgia, N Caucasus, Mtskheta-Mtianeti (Khevsureti), Mutso village, 1570 m, 45.20378°N, 42.62°E, 1 June 2017, *N. Silakadze & al. 70a* (B 10 1052346), PP004778, PP004453, PP004127; CAM1169b [DB 41685]: Georgia, N Caucasus, Mtskheta-Mtianeti (Khevsureti), Mutso village, 1500 m, 45.20465°N, 42.62017°E, 1 June 2017, *N. Silakadze & al. 70b* (B 10 1052347), PP004779, PP004454, PP004128; CAM1169c [DB 41686]: Georgia, N Caucasus, Mtskheta-Mtianeti (Khevsureti), Mutso village, 1520 m, 45.20511°N, 42.62004°E, 1 June 2017, *N. Silakadze & al. 70c* (B 10 1052348), PP004780, PP004455, PP004129; CAM1169d [DB 41687]: *N. Silakadze & al. 70d* (B 10 1052349), PP004781, PP004456, PP004130, from the same population as *70c*; CAM1169e [DB 41688]: Georgia, N Caucasus, Mtskheta-Mtianeti (Khevsureti), Mutso village, 1480 m, 45.20398°N, 42.61689°E, 1 June 2017, *N. Silakadze & al. 70e* (B 10 1052350), PP004782, PP004457, PP004131; CAM1170a [DB 41689]: Georgia, N Caucasus, Mtskheta-Mtianeti (Khevsureti), between Ardoti and Mutso villages, 1600 m, 45.21127°N, 42.59964°E, 1 June 2017, *N. Silakadze & al. 71a* (B 10 1052351), PP004783, PP004458, PP004132; CAM1170b [DB 41690]: *N. Silakadze & al. 71b* (B 10 1052352), PP004784, PP004459, PP004133, from the same population as *71a*; CAM1170c [DB 41691]: Georgia, N Caucasus, Mtskheta-Mtianeti (Khevsureti), between Ardoti and Mutso villages, 1700 m, 45.19035°N, 42.57224°E, 1 June 2017, *N. Silakadze & al. 71c* (B 10 1052353), PP004785, PP004460, PP004134; CAM1170d [DB 41692]: Georgia, N Caucasus, Mtskheta-Mtianeti (Khevsureti), between Ardoti and Mutso villages, 1710 m, 45.19025°N, 42.57221°E, 1 June 2017, *N. Silakadze & al. 71d* (B 10 1052354), PP004786, PP004461, PP004135; CAM1170e [DB 41693]: Georgia, N Caucasus, Mtskheta-Mtianeti (Khevsureti), between Ardoti and Mutso villages, 1910 m, 45.21297°N, 42.60002°E, 1 June 2017, *N. Silakadze & al. 71e* (B 10 1052355), PP004787, PP004462, PP004136; CAM1200 [DB 41759]: Georgia, N Caucasus, Kakheti (Tusheti), Akhmeta district, Abano Pass, 1620 m, 45.49331°N, 42.2392°E, 27 June 2017, *N. Silakadze & al. 101* (B 10 1052421), PP004837, PP004513, PP004187; CAM1201b [DB 41761]: Georgia, N Caucasus, Kakheti (Tusheti), Akhmeta district, Kvavlo village, 2120 m, 45.58375°N, 42.44753°E, 28 June 2017, *N. Silakadze & al. 102b* (B 10 1052423), PP004838, PP004514, PP004188; CAM1202a [DB 41762]: Georgia, N Caucasus, Kakheti (Tusheti), Akhmeta district, Dano village, 2180 m, 45.56667°N, 42.45°E, 28 June 2017, *N. Silakadze & al. 103a* (B 10 1052424), PP004839, PP004515, PP004189; CAM1202b [DB 41763]: *N. Silakadze & al. 103b* (B 10 1052425), PP004840, PP004516, PP004190, from the same population as *103a*; CAM1203a [DB 41764]: Georgia, N Caucasus, Kakheti (Tusheti), Akhmeta district, road to Chesho village, 1970 m, 45.54361°N, 42.4755°E, 28 June 2017, *N. Silakadze & al. 104a* (B 10 1052426), PP004841, PP004517, PP004191; CAM1203b [DB 41765]: *N. Silakadze & al. 104b* (B 10 1052427), PP004842, PP004518, PP004192, from the same population as *104a*; CAM1203c [DB 41766]: *N. Silakadze & al. 104c* (B 10 1052428), PP004843, PP004519, PP004193, from the same population as *104a*; CAM1203d [DB 41767]: *N. Silakadze & al. 104d* (B 10 1052429), PP004844, PP004520, PP004194, from the same population as *104a*; CAM1204a [DB 41768]: Georgia, N Caucasus, Kakheti (Tusheti), Akhmeta district, Abano Pass, 2410 m, 45.49041°N, 42.26998°E, 29 June 2017, *N. Silakadze & al. 105a* (B 10 1052430), PP004845, PP004521, PP004195; CAM1204b [DB 41769]: Georgia, N Caucasus, Kakheti (Tusheti), Akhmeta district, Abano Pass, 2370 m, 45.49472°N, 42.26881°E, 29 June 2017, *N. Silakadze & al. 105b* (B 10 1052431), PP004846, PP004522, PP004196; CAM1204c [DB 41770]: *N. Silakadze & al. 105c* (B 10 1052432), PP004847, PP004523, PP004197, from the same population as *105b*; CAM1204d [DB 41771]: *N. Silakadze & al. 105d* (B 10 1052433), PP004848, PP004524, PP004198, from the same population as *105b*; CAM1229 [DB 41826]: Russian Federation, Dagestan, N Caucasus, Akhtynsky District, Kurush village, 23 July 1986, *Y. L. Menitsky & al.* s.n. (ERE 69616), PP004907, PP004559, PP004233; CAM1231 [DB 41828]: Azerbaijan, Oghuz district, Bash-Dashagil village, 28 June 1973, *J. Hashimov* s.n. (ERE 66955), PP004908, PP004560, PP004234; CAM1274 [DB 41867]: Georgia, N Caucasus, Kakheti (Tusheti), Akhmeta district, Parsma village, 2000 m, 16 August 1987, *D. Chelidze & Sh. Shetekauri* s.n. (TBI 1038261), PP004899, PP004594, PP004267; CAM1291 [DB 41884]: Georgia, N Caucasus, Kakheti (Tusheti), Akhmeta district, Etelta village, the river Gometsris Alazani, 2000 m, 7 August 1986, *Sh. Shetekauri* s.n. (TBI 1038260), PP004935, PP004608, PP004282.

*Campanulaarmazica* Kharadze: CAM1078b [DB 41321]: Georgia, S Caucasus, Mtskheta-Mtianeti, Mtskheta district, between Mtskheta and Karsan, 790 m, 44.70372°N, 41.83316°E, 24 June 2016, *N. Silakadze & Z. Janiashvili 1b* (B 10 1052201), PP004659, PP004334, PP004008; CAM1175a [DB 41700]: Georgia, S Caucasus, Mtskheta-Mtianeti, Mtskheta district, between Mtskheta and Karsan, 750 m, 44.70372°N, 41.83316°E, 3 June 2017, *N. Silakadze & al. 76a* (B 10 1052362), PP004791, PP004466, PP004140; CAM1175b [DB 41701]: *N. Silakadze & al. 76b* (B 10 1052363), PP004792, PP004467, PP004141, from the same population as *76a*.

*Campanulaaucheri* A. DC.: CAM1079a [DB 41325]: Georgia, S Caucasus, Kvemo Kartli, Tsalka district, Cholmani village, Didi Kldekari Mountain, 2040 m, 44.21004°N, 41.74328°E, 29 June 2016, *N. Silakadze 2a* (B 10 1052202), PP004660, PP004335, PP004009; CAM1079b [DB 41326]: *N. Silakadze 2b* (B 10 1052203), PP004661, PP004336, PP004010, from the same population as *2a*; CAM1135a [DB 41622]: Georgia, S Caucasus, Samckhe-Javakheti, Borjomi district, Bakuriani, Tskhratskaro Pass, 2400 m, 43.51897°N, 41.69375°E, 10 August 2016, *N. Silakadze 62a* (B 10 1052306), PP004740, PP004415, PP004089; CAM1135b [DB 41623]: *N. Silakadze 62b* (B 10 1052307), PP004741, PP004416, PP004090, from the same population as *62a*; CAM1135c [DB 41624]: *N. Silakadze 62c* (B 10 1052308), PP004742, PP004417, PP004091, from the same population as *62a*; CAM1135d [DB 41625]: Georgia, S Caucasus, Samckhe-Javakheti, Borjomi district, Bakuriani, Tskhratskaro Pass, 2210 m, 43.51307°N, 41.70018°E, 10 August 2016, *N. Silakadze 62d* (B 10 1052309), PP004743, PP004418, PP004092; CAM1135e [DB 41626]: *N. Silakadze 62e* (B 10 1052310), PP004744, PP004419, PP004093, from the same population as *62d*; CAM1143a [DB 41636]: Georgia, S Caucasus, Samckhe-Javakheti, Borjomi district, Tsikhisjvari village, Kodiani Mountain, 2410 m, 43.36113°N, 41.72723°E, 11 August 2016, *N. Silakadze 68a* (B 10 1052320), PP004752, PP004427, PP004101 PP004101 ; CAM1143c [DB 41638]: Georgia, S Caucasus, Samckhe-Javakheti, Borjomi district, Tsikhisjvari village, Kodiani Mountain, 2480 m, 43.36077°N, 41.72632°E, 11 August 2016, *N. Silakadze 68c* (B 10 1052322), PP004753, PP004428, PP004102; CAM1143e [DB 41640]: Georgia, S Caucasus, Samckhe-Javakheti, Borjomi district, Tsikhisjvari village, Kodiani Mountain, 2500 m, 43.36038°N, 41.726°E, 11 August 2016, *N. Silakadze 68e* (B 10 1052324), PP004754, PP004429, PP004103; CAM1143g [DB 41642]: Georgia, S Caucasus, Samckhe-Javakheti, Borjomi district, Tsikhisjvari village, Kodiani Mountain, 2510 m, 43.3602°N, 41.72592°E, 11 August 2016, *N. Silakadze 68g* (B 10 1052326), PP004755, PP004430, PP004104; CAM1143h [DB 41643]: Georgia, S Caucasus, Samckhe-Javakheti, Borjomi district, Tsikhisjvari village, Kodiani Mountain, 2540 m, 43.3604°N, 41.72565°E, 11 August 2016, *N. Silakadze 68h* (B 10 1052327), PP004756, PP004431, PP004105; CAM1143j [DB 41645]: *N. Silakadze 68j* (B 10 1149579), PP004757, PP004432, PP004106, from the same population as *68h*; CAM1143k [DB 41646]: Georgia, S Caucasus, Samckhe-Javakheti, Borjomi district, Tsikhisjvari village, Kodiani Mountain, 2570 m, 43.36072°N, 41.7253°E, 11 August 2016, *N. Silakadze 68k* (B 10 1052330), PP004758, PP004433, PP004107; CAM1179a [DB 41716]: Georgia, N Caucasus, Mtskheta-Mtianeti (Kazbegi), daba Stepantsminda, Sioni village, Kabarjina Mountain, 2190 m, 44.58766°N, 42.57445°E, 8 June 2017, *N. Silakadze & K. E. Jones 80a* (B 10 1052378), PP004804, PP004479, PP004153; CAM1179b [DB 41717]: *N. Silakadze & K. E. Jones 80b* (B 10 1052379), PP004805, PP004480, PP004154, from the same population as *80a*; CAM1180 [DB 41719]: Georgia, N Caucasus, Mtskheta-Mtianeti (Kazbegi), daba Stepantsminda, Sioni village, Kabarjina Mountain, 2190 m, 44.59061°N, 42.57493°E, 8 June 2017, *N. Silakadze & K. E. Jones 81* (B 10 1052381), PP004807, PP004482, PP004156; CAM1187a [DB 41728]: Georgia, S Caucasus, Samckhe-Javakheti, Borjomi district, Bakuriani, Tskhratskaro Pass, 2120 m, 43.50328°N, 41.70553°E, 13 June 2017, *N. Silakadze & K. E. Jones 88a* (B 10 1052390), PP004814, PP004490, PP004164; CAM1187c [DB 41730]: Georgia, S Caucasus, Samckhe-Javakheti, Borjomi district, Bakuriani, Tskhratskaro Pass, 2380 m,43.51889°N, 41.69384°E, 13 June 2017, *N. Silakadze & K. E. Jones 88c* (B 10 1052392), PP004815, PP004815, PP004815; CAM1188 [DB 41731]: Georgia, S Caucasus, Samckhe-Javakheti, Borjomi district, Tsikhisjvari village, Kodiani Mountain, 2550 m, 43.36107°N, 41.72514°E, 13 June 2017, *N. Silakadze & K. E. Jones 89*(B 10 1052393), PP004816, PP004492, PP004166; CAM1190b [DB 41734]: Georgia, S Caucasus, Samckhe-Javakheti, daba Aspindza, Vardzia, 1260 m, 43.27569°N, 41.37382°E, 14 June 2017, *N. Silakadze & K. E. Jones 91b* (B 10 1052396), PP004817, PP004493, PP004167; CAM1190c [DB 41735]: *N. Silakadze & K. E. Jones 91c* (B 10 1052397), PP004818, PP004494, PP004168, from the same population as *91b*; CAM1190d [DB 41736]: *N. Silakadze & K. E. Jones 91d* (B 10 1052398), PP004819, PP004495, PP004169, from the same population as *91b*; CAM1190e [DB 41737]: *N. Silakadze & K. E. Jones 91e* (B 10 1052399), PP004820, PP004496, PP004170, from the same population as *91b*; CAM1194a [DB 41742]: Georgia, S Caucasus, Samckhe-Javakheti, Akhalkalaki district, Abuli Mountain, 2370 m, 43.65961°N, 41.40294°E, 16 June 2017, *N. Silakadze & K. E. Jones 95a* (B 10 1052404), PP004822, PP004498, PP004172; CAM1194b [DB 41743]: Georgia, S Caucasus, Samckhe-Javakheti, Akhalkalaki district, Abuli Mountain, 2370 m, 43.65981°N, 41.40407°E, 16 June 2017, *N. Silakadze & K. E. Jones 95b* (B 10 1052405), PP004823, PP004499, PP004173; CAM1194c [DB 41744]: Georgia, S Caucasus, Samckhe-Javakheti, Akhalkalaki district, Abuli Mountain, 2390 m, 43.65968°N, 41.40417°E, 16 June 2017, *N. Silakadze & K. E. Jones 95c* (B 10 1052406), PP004824, PP004500, PP004174; CAM1196a [DB 41747]: Georgia, S Caucasus, Kvemo kartli, Manglisi district, Birtvisi Mountain, 1010 m, 44.54022°N, 41.604°E, 21 June 2017, *N. Silakadze & al. 97a* (B 10 1052409), PP004826, PP004502, PP004176; CAM1196b [DB 41748]: *N. Silakadze & al. 97b* (B 10 1052410), PP004827, PP004503, PP004177, from the same population as *97a*; CAM1196c [DB 41749]: *N. Silakadze & al. 97c* (B 10 1052411), PP004828, PP004504, PP004178, from the same population as *97a*; CAM1196d [DB 41750]: *N. Silakadze & al. 97d* (B 10 1052412), PP004829, PP004505, PP004179, from the same population as *97a*; CAM1197b [DB 41752]: Georgia, S Caucasus, Kvemo kartli, Tsalka district, Akhalshenari village, 1680 m, 44.07125°N, 41.6725°E, 22 June 2017, *N. Silakadze & al. 98b* (B 10 1052414), PP004830, PP004506, PP004180; CAM1197c [DB 41753]: *N. Silakadze & al. 98b* (B 10 1052415), PP004831, PP004507, PP004181, from the same population as *98b*; CAM1198 [DB 41754]: Georgia, S Caucasus, Kvemo kartli, Tsalka district, Kaburi village, 2000 m, 44.01223°N, 41.71483°E, 22 June 2017, *N. Silakadze & al. 99* (B 10 1052416), PP004832, PP004508, PP004182; CAM1199a [DB 41755]: Georgia, S Caucasus, Kvemo kartli, Tsalka district, Khachkoi village, Arjevani Mountain, 1980 m, 43.97305°N, 41.71371°E, 22 June 2017, *N. Silakadze & al. 100a* (B 10 1052417), PP004833, PP004509, PP004183; CAM1199b [DB 41756]: *N. Silakadze & al. 100b* (B 10 1052418), PP004834, PP004510, PP004184, from the same population as *100a*; CAM1199c [DB 41757]: *N. Silakadze & al. 100c* (B 10 1052419), PP004835, PP004511, PP004185, from the same population as *100a*; CAM1199d [DB 41758]: *N. Silakadze & al. 100d*(B 10 1052420), PP004836, PP004512, PP004186, from the same population as *100a*; CAM1222a [DB 41816]: Armenia, S Caucasus, Gegharkunik Province, Lake Sevan, Surroundings of Sevan lake, on the old road, 1990 m, 44.99379°N, 40.57617°E, 22 August 2017, *N. Silakadze & M. Oganesian 123a* (B 10 1052478), PP004878, PP004555, PP004229; CAM1222b [DB 41817]: *N. Silakadze & M. Oganesian 123b* (B 10 1052479), PP004879, PP004556, PP004230, from the same population as *123a*; CAM1223b [DB 41819]: Armenia, S Caucasus, Gegharkunik Province, Gegha Mountain, 2480 m, 44.91523°N, 40.42012°E, 22 August 2017, *N. Silakadze & M. Oganesian 124b* (B 10 1052481), PP004880, PP004557, PP004231; CAM1233 [DB 41829]: Azerbaijan, S Caucasus, Kalbajar raion, Surroundings Istisu village, 19 June 1985, *Yu. L. Menitsky* s.n. (ERE 66880), PP004881, PP004561, PP004235; CAM1234 [DB 41830]: Turkey, S Caucasus, Province Kars, South-West side of Kisir dag, 2700 m, 3 July 1957, *Davis & Hedge D30499B* (ERE 56333), PP004909, PP004562, PP004236; CAM1252 [DB 41848]: Armenia, S Caucasus, Kotayk Province, Mountain Arayi Lerr, surroundings of the Monastery Gharghavank (Spitakavor Monastery), 6 July 1989, *E. Gabrielian & M. Oganesian* s.n. (ERE 141517), PP004917, PP004579, PP004253; CAM1254 [DB 41850]: Armenia, S Caucasus, Shirak Province, Amasia district, 1 km W of village Bandivan, 1850 m, 43.8°N, 40.96666°E, 21 June 2003, *M. Oganezian & al.* s.n. (ERE 152386), PP004891, PP004580, PP004254; CAM1266 [DB 41859]: Georgia, S Caucasus, Samckhe-Javakheti, Tetrobi range, 24 July 1967, *L. Khintibidze* s.n. (TBI 1036646), PP004895, PP004587, PP004261; CAM1310 [DB 41889]: Iran, S Caucasus, In monte Chalil Kuh prope Razhan [Rajan Khalil, Mountain], 2600-3200 m, 2 July 1974, *W. Rechinger & J. Renz 48816* (B 10 1015488), PP004948, PP004622, PP004296; CAM1311 [DB 41890]: Iran, S Caucasus, West Azerbaijan Province, Urma, Darband [Urmia, darband, silvaneh], 2000 m, 3 June 1994, *S. Narimisa 410* (B 10 0626006), PP004949, PP004623, PP004297.

*Campanulabellidifolia* Adams: CAM1172b [DB 41696]: Georgia, N Caucasus, Mtskheta-Mtianeti (Khevsureti), Shatili village, 1570 m, 45.14587°N, 42.67244°E, 2 June 2017, *N. Silakadze & al. 73b* (B 10 1052358), PP004788, PP004463, PP004137; CAM1172c [DB 41697]: *N. Silakadze & al. 73c* (B 10 1052359), PP004789, PP004464, PP004138, from the same population as *73b*; CAM1177a [DB 41704]: Georgia, N Caucasus, Mtskheta-Mtianeti (Kazbegi), daba Stepantsminda, Tsdo village, 1660 m, 44.63317°N, 42.68351°E, 7 June 2017, *N. Silakadze & K. E. Jones 78a* (B 10 1052366), PP004793, PP004468, PP004142; CAM1177b [DB 41705]: Georgia, N Caucasus, Mtskheta-Mtianeti (Kazbegi), daba Stepantsminda, Tsdo village, 1670 m, 44.63321°N, 42.68466°E, 7 June 2017, *N. Silakadze & K. E. Jones 78b* (B 10 1052367), PP004794, PP004469, PP004143; CAM1177c [DB 41706]: *N. Silakadze & K. E. Jones 78c* (B 10 1052368), PP004795, PP004470, PP004144, from the same population as *78b*; CAM1177d [DB 41707]: *N. Silakadze & K. E. Jones 78d* (B 10 1052369), PP004796, PP004471, PP004145, from the same population as *78b*; CAM1177e [DB 41708]: *N. Silakadze & K. E. Jones 78e* (B 10 1052370), PP004797, PP004472, PP004146, from the same population as *78b*; CAM1177h [DB 41711]: Georgia, N Caucasus, Mtskheta-Mtianeti (Kazbegi), daba Stepantsminda, Dariali gorge, 1280 m, 44.62981°N, 42.7387°E, 7 June 2017, *N. Silakadze & K. E. Jones 78h* (B 10 1052373), PP004799, PP004474, PP004148; CAM1177i [DB 41712]: *N. Silakadze & K. E. Jones 78i* (B 10 1052374), PP004800, PP004475, PP004149, from the same population as *78h*; CAM1177j [DB 41713]: *N. Silakadze & K. E. Jones 78j* (B 10 1052375), PP004801, PP004476, PP004150, from the same population as *78h*; CAM1177k [DB 41714]: *N. Silakadze & K. E. Jones 78k* (B 10 1052376), PP004802, PP004477, PP004151, from the same population as *78h*; CAM1178 [DB 41715]: Georgia, N Caucasus, Mtskheta-Mtianeti (Kazbegi), daba Stepantsminda, Gveleti village, Devdoraki gorge, 1810 m, 44.59435°N, 42.7246°E, 7 June 2017, *N. Silakadze & K. E. Jones 79* (B 10 1052377), PP004803, PP004478, PP004152; CAM1179c [DB 41718]: Georgia, N Caucasus, Mtskheta-Mtianeti (Kazbegi), daba Stepantsminda, Sioni village, Kabarjina Mountain, 2190 m, 44.58766°N, 42.57445°E, 8 June 2017, *N. Silakadze & K. E. Jones 80c* (B 10 1052380), PP004806, PP004481, PP004155; CAM1182 [DB 41722]: Georgia, N Caucasus, Mtskheta-Mtianeti (Kazbegi), daba Stepantsminda, Sioni village, Kabarjina Mountain, 2110 m, 44.58824°N, 42.57915°E, 8 June 2017, *N. Silakadze & K. E. Jones 83* (B 10 1052384), PP004809, PP004484, PP004158; CAM1184 [DB 41725]: Georgia, N Caucasus, Mtskheta-Mtianeti (Kazbegi), daba Stepantsminda, Gergeti Church, Sabertse, 2440 m, 44.59739°N, 42.66365°E, 9 June 2017, *N. Silakadze & K. E. Jones 85* (B 10 1052387), PP004904, PP004487, PP004161; CAM1186 [DB 41727]: Georgia, N Caucasus, Mtskheta-Mtianeti (Kazbegi), daba Stepantsminda, Elia Mountain, 1880 m, 44.65625°N, 42.65376°E, 9 June 2017, *N. Silakadze & K. E. Jones 87* (B 10 1052389), PP004813, PP004489, PP004163; CAM1292 [DB 41740]: Georgia, N Caucasus, Mtskheta-Mtianeti (Kazbegi), Devdoraki gorge, 1 August 1967, *E. Khutsishvili* s.n. (B 10 1052402), PP004936, PP004609, PP004283.

*Campanulabesenginica* Fomin: CAM1236 [DB 41832]: Russian Federation, N Caucasus, Kabardino-Balkaria, Sovet raion, 5 km from Bezengi village to north, 6 June 1985, *Yu. L. Menitsky* s.n. (ERE 70791), PP004911, PP004564, PP004238; CAM1261 [DB 41854]: Russian Federation, N Caucasus, Kabardino-Balkaria, Bezengi glacier, July 1893, *I. J. Akinfiev* s.n. (TBI 1033286), PP004920, PP004584, PP004258.

*Campanulabiebersteiniana* Roem. & Schult.: CAM1070 [DB 41299]: Russian Federation, N Caucasus, Republic of Adygea, Maikopskiy rayon, Lagonaki plateau, just S of lake Psenodakh, 1950-2000 m, 39.9°N, 44.00833°E, 29 July 2015, *E. von Raab-Straube & al. 6932* (B 10 1052542), PP004652, PP004327, PP004001; CAM1072c [DB 41303]: Russian Federation, N Caucasus, Republic of Adygea, Maikopskiy rayon, Lagonaki plateau, near lake Psenodakh, at the N foot of Mt. Pshekho-Su, 1950-2000 m, 39.9°N, 44.00833°E, 30 July 2015, *E. von Raab-Straube & al. 6941c* (B 10 1052545), PP004654, PP004329, PP004003; CAM1072g [DB 41307]: *E. von Raab-Straube & al. 6941g* (B 10 1052545), PP004655, PP004330, PP004004, from the same population as *6941c*; CAM1074a [DB 41309]: Russian Federation, N Caucasus, Republic of Adygea, Maikopskiy rayon, Lagonaki plateau, just S of Fisht-Oshtenskiy pereval [= Pass between Mts. Fisht and Oshten], 2200 m, 39.91388°N, 43.99055°E, 30 July 2015, *E. von Raab-Straube & al. 7001a* (B 10 1052547), PP004656, PP004331, PP004005; CAM1074f [DB 41314]: *E. von Raab-Straube & al. 7001f* (B 10 1052547), PP004657, PP004332, PP004006, from the same population as *7001a*; CAM1084a [DB 41358]: Georgia, N Caucasus, Kakheti (Tusheti), Akhmeta district, Abano Pass, 2310 m, 45.512033°N, 42.28488°E, 6 July 2016, *N. Silakadze & al. 7a* (B 10 1052208), PP004663, PP004338, PP004012; CAM1131b [DB 41585]: Georgia, N Caucasus, Samegrelo-Zemo Svaneti (Zemo Svaneti), Mestia district, Mestia-Koruldi Lake, 2730 m, 42.70793°N, 43.08657°E, 28 July 2016, *N. Silakadze & al. 56b* (B 10 1052295), PP004731, PP004406, PP004080; CAM1131t [DB 41603]: Georgia, N Caucasus, Samegrelo-Zemo Svaneti (Zemo Svaneti), Mestia district, Mestia-Koruldi Lake, 2750 m, 42.703°N, 43.08707°E, 28 July 2016, *N. Silakadze & al. 56t* (B 10 1052296), PP004732, PP004407, PP004081; CAM1133b [DB 41607]: Georgia, N Caucasus, Samegrelo-Zemo Svaneti (Zemo Svaneti), Mestia district, Tetnuldi Mountain, 3040 m, 42.91712°N, 43.0287°E, 30 July 2016, *N. Silakadze & al. 60b* (B 10 1052299), PP004734, PP004409, PP004083; CAM1133d [DB 41609]: *N. Silakadze & al. 60d* (B 10 1052301), PP004735, PP004410, PP004084, from the same population as *60b*; CAM1133e [DB 41610]: Georgia, N Caucasus, Samegrelo-Zemo Svaneti (Zemo Svaneti), Mestia district, Tetnuldi Mountain, 3070 m, 42.91907°N, 43.02922°E, 30 July 2016, *N. Silakadze & al. 60e* (B 10 1052302), PP004736, PP004411, PP004085; CAM1133k [DB 41616]: Georgia, N Caucasus, Samegrelo-Zemo Svaneti (Zemo Svaneti), Mestia district, Tetnuldi Mountain, 3190 m, 42.9172°N, 43.03205°E, 30 July 2016, *N. Silakadze & al. 60k* (B 10 1052304), PP004737, PP004412, PP004086; CAM1173 [DB 41698]: Georgia, N Caucasus, Mtskheta-Mtianeti (Khevsureti), Datvijvari gorge, 1970 m, 45.10094°N, 42.5773°E, 2 June 2017, *N. Silakadze & al. 74* (B 10 1052360), PP004790, PP004465, PP004139; CAM1181b [DB 41721]: Georgia, N Caucasus, Mtskheta-Mtianeti (Kazbegi), daba Stepantsminda, Sioni village, Kabarjina Mountain, 2230 m, 44.59172°N, 42.57534°E, 8 June 2017, *N. Silakadze & K. E. Jones 82b* (B 10 1052383), PP004808, PP004483, PP004157; CAM1183a [DB 41723]: Georgia, N Caucasus, Mtskheta-Mtianeti (Kazbegi), daba Stepantsminda, Gergeti Church, Sabertse, 3020 m, 44.55905°N, 42.66031°E, 9 June 2017, *N. Silakadze & K. E. Jones 84a* (B 10 1052385), PP004810, PP004485, PP004159; CAM1185 [DB 41726]: Georgia, N Caucasus, Mtskheta-Mtianeti (Kazbegi), daba Stepantsminda, Gergeti Church, 2260 m, 44.61147°N, 42.66489°E, 9 June 2017, *N. Silakadze & K. E. Jones 86* (B 10 1052388), PP004812, PP004488, PP004162; CAM1298 [DB 40121]: Russian Federation, N Caucasua, Kabardino-Balkaria, Mt. Elbrus, S slopes, E of lift station “Mir”, 3450 m, 42.46472°N, 43.28944°E, 20 July 2019, *G. Parolly & al. 15769* (B 10 1118233B), PP004903, PP004615, PP004289.

*Campanulachamissonis* Fed.: CAM1239 [DB 41835]: Russia, Sakhalin, Karakulchan range, 500 m, 26 July 1968, *G. Pomomarczuk* s.n. (ERE 55255), PP004882, PP004566, PP004240.

*Campanulaciliata* Steven: CAM1038f [DB 41220]: Russian Federation, N Caucasus, Karachay-Cherkessia, Karachayevsky rayon, Skalistyy khrebet, SE slopes of Mt. Gumbashi, 2000 m, 42.20556°N, 43.77972°E, 25 June 2015, *T. Borsch & al. 5790f* (B 10 1052498), PP004631, PP004306, PP003980; CAM1038o [DB 41229]: *T. Borsch & al. 5790o* (B 10 1052498), PP004632, PP004307, PP003981, from the same population as *5790f*; CAM1039c [DB 41232]: Russian Federation, N Caucasus, Karachay-Cherkessia, Karachayevsky rayon, Skalistyy khrebet, SE slopes of Mt. Gumbashi, 2100 m, 42.20556°N, 43.77972°E, 25 June 2015, *T. Borsch & al. 5802c* (B 10 1052501), PP004633, PP004308, PP003982; CAM1039n [DB 41243]: *T. Borsch & al. 5802n* (B 10 1052501), PP004634, PP004309, PP003983, from the same population as *5802c*; CAM1039s [DB 41248]: *T. Borsch & al. 5802s* (B 10 1052501), PP004635, PP004310, PP003984, from the same population as *5802c*; CAM1067c [DB 41296]: Russian Federation, N Caucasus, Republic of Adygea, Maikopskiy rayon, Lagonaki plateau, N slopes of Mt. Pshekho-Su, ca. 1 km S of lake Psenodakh, 2200 m, 39.895°N, 44.00194°E, 30 July 2015, *E. von Raab-Straube & al. 6969c* (B 10 1052538), PP004649, PP004324, PP003998; CAM1183b [DB 41724]: Georgia, N Caucasus, Mtskheta-Mtianeti (Kazbegi), daba Stepantsminda, Gergeti Church, Sabertse, 3020 m, 44.55905°N, 42.66031°E, 9 June 2017, *N. Silakadze & K. E. Jones 84b* (B 10 1052386), PP004811, PP004486, PP004160; CAM1240 [DB 41836]: Russian Federation, N Caucasus, Krasnodar Krai, Oshten Mountain, 2500-2700 m, 14 July 1981, *Yu. L. Menitsky & al. 414* (ERE 66937), PP004883, PP004567, PP004241; CAM1241 [DB 41837]: Russian Federation, N Caucasus, Kabardino-Balkaria, Zapadnyy Kinzhal mountain, 30 July 1981, *Yu. L. Menitsky & al. 431* (ERE 66938), PP004884, PP004568, PP004242; CAM1243 [DB 41839]: Azerbaijan, N Caucasus, Qusar, The right side of river Kusarchay, village Laza, Karadan Mountain, 12 July 1978, *Yu. L. Menitsky & al. 105* (ERE 70849), PP004886, PP004570, PP004244.

*Campanulacircassica* Fomin: CAM1130e [DB 41570]: Georgia, N Caucasus, Samegrelo-Zemo Svaneti (Zemo Svaneti), Mestia district, Mestia-Koruldi Lake, 2630 m, 42.7113°N, 43.08155°E, 28 July 2016, *N. Silakadze & al. 55e* (B 10 1052280), PP004721, PP004396, PP004070; CAM1130k [DB 41576]: *N. Silakadze & al. 55k* (B 10 1052286), PP004724, PP004399, PP004073, from the same population as *55e*; CAM1130q [DB 41582]: Georgia, N Caucasus, Samegrelo-Zemo Svaneti (Zemo Svaneti), Mestia district, Mestia-Koruldi Lake, 2750 m, 42.703°N, 43.08707°E, 28 July 2016, *N. Silakadze & al. 55q* (B 10 1052292), PP004729, PP004404, PP004078; CAM1130r [DB 41583]: Georgia, N Caucasus, Samegrelo-Zemo Svaneti (Zemo Svaneti), Mestia district, Mestia-Koruldi Lake, 2750 m, 42.70257°N, 43.08717°E, 28 July 2016, *N. Silakadze & al. 55r* (B 10 1052293), PP004730, PP004405, PP004079; CAM1242 [DB 41838]: Russian Federation, N Caucasus, Krasnodar Krai, Adler raion, Cochi, surroundings village Krasnaya Polyana, Aibga Ridge, 23 July 2011, *B. S. Tuniev* s.n. (ERE 80037), PP004885 PP004569, PP004243.

Campanulaaff.collina Sims: CAM1062 [DB 41289]: *E. von Raab-Straube & al. 6830* (B 10 1052532), PP004645, PP004320, PP003994, from the same population as *6832*; CAM1037 [DB 41214]: *T. Borsch & al. 5812* (B 10 1052495), PP004630, PP004305, PP003979, from the same population as *5810*; CAM1071 [DB 41300]: Russian Federation, N Caucasus, Republic of Adygea, Maikopskiy rayon, Lagonaki plateau, near lake Psenodakh, at the N foot of Mt. Pshekho-Su, 1950-2000 m, 39.9°N, 44.00833°E, 30 July 2015, *E. von Raab-Straube & al. 6935* (B 10 1052543), PP004653, PP004328, PP004002; CAM1076 [DB 41316]: Russian Federation, N Caucasus, Republic of Adygea, Maikopskiy rayon, Lagonaki plateau, N slopes of Mt. Pshekho-Su, ca. 1 km S of lake Psenodakh, 2200 m, 39.895°N, 44.00194°E, 30 July 2015, *E. von Raab-Straube & al. 6981* (B 10 1052549), PP004658, PP004333, PP004007; CAM1081a [DB 41337]: Georgia, S Caucasus, Kvemo Kartli, Tsalka district, Cholmani village, Didi Kldekari Mountain, 1960 m, 44.2051°N, 41.74266°E, 29 June 2016, *N. Silakadze & al. 4a* (B 10 1052205), PP004662, PP004337, PP004011; CAM1088 [DB 41385]: Georgia, N Caucasus, Kakheti (Tusheti), Akhmeta district, Dano village, 2150 m, 45.56757°N, 42.45046°E, 7 July 2016, *N. Silakadze & al. 11* (B 10 1052212), PP004666, PP004341, PP004015; CAM1094 [DB 41417]: Georgia, N Caucasus, Kakheti (Tusheti), Akhmeta district, Dochu village, 2230 m, 45.5544°N, 42.39217°E, 8 July 2016, *N. Silakadze & al. 19* (B 10 1052220), PP004674, PP004349, PP004023; CAM1101 [DB 41434]: Georgia, N Caucasus, Kakheti (Tusheti), Akhmeta district, Dartlo village, 1910 m, 45.58929°N, 42.4276°E, 9 July 2016, *N. Silakadze & al. 26* (B 10 1052229), PP004682, PP004357, PP004031; CAM1109 [DB 41480]: Georgia, N Caucasus, Kakheti (Tusheti), Akhmeta district, Abano Pass, 2290 m, 45.52758°N, 42.29441°E, 10 July 2016, *N. Silakadze & al. 34* (B 10 1052239), PP004690, PP004365, PP004039; CAM1116 [DB 41504]: Georgia, N Caucasus, Racha-Lechkhumi and Kvemo Svaneti (Racha), Oni district, Shkmeri village, 1650 m, 43.40138°N, 42.48907°E, 21 July 2016, *N. Silakadze & al. 41* (B 10 1052248), PP004697, PP004372, PP004046; CAM1121 [DB 41509]: Georgia, N Caucasus, Racha-Lechkhumi and Kvemo Svaneti (Lechkhumi), Tsageri district, Khvamli Mountain, 1880 m, 42.72087°N, 42.50805°E, 22 July 2016, *N. Silakadze & al. 45* (B 10 1052253), PP004700, PP004375, PP004049; CAM1129 [DB 41565]: Georgia, N Caucasus, Samegrelo-Zemo Svaneti (Samegrelo), Martvili district, Taleri village, Chegola Mountain, 2430 m, 42.42293°N, 42.77655°E, 26 July 2016, *N. Silakadze & al. 54* (B 10 1052275), PP004720, PP004395, PP004069; CAM1132 [DB 41605]: Georgia, N Caucasus, Samegrelo-Zemo Svaneti (Zemo Svaneti), Mestia district, Mestia-Koruldi Lake, 2480 m, 42.71327°N, 43.07747°E, 28 July 2016, *N. Silakadze & al. 59* (B 10 1052297), PP004733, PP004408, PP004082; CAM1136 [DB 41627]: Georgia, S Caucasus, Samckhe-Javakheti, Borjomi district, Bakuriani, Tskhratskaro Pass, 2400 m, 43.51897°N, 41.69375°E, 10 August 2016, *N. Silakadze 2* (B 10 1052311), PP004745, PP004420, PP004094; CAM1137 [DB 41628]: *N. Silakadze 63-2* (B 10 1052312), PP004746, PP004421, PP004095, from the same population as *63-1*; CAM1141 [DB 41634]: Georgia, S Caucasus, Samckhe-Javakheti, Borjomi district, Tsikhisjvari village, Kodiani Mountain, 2390 m, 43.36105°N, 41.72778°E, 11 August 2016, *N. Silakadze 66* (B 10 1052318), PP004750, PP004425, PP004099; CAM1142 [DB 41635]: *N. Silakadze 67* (B 10 1052319), PP004751, PP004426, PP004100, from the same population as *66*; CAM1296 [DB 40117]: Russian Federation, N Caucasua, Kabardino-Balkari-a, on Mountain road between Chegem and Baksan valley (“Aktaprak”), 1900 m, 43.08889°N, 43.40694°E, 19 July 2019, *G. Parolly & al. 15730* (B 10 1118230), PP004940, PP004613, PP004287. CAM1297 [DB 40119]: Russian Federation, N Caucasua, Kabardino-Balkaria, Baksan valley, between Azau village and Azau waterfall, 2360 m, 42.47528°N, 43.265°E, 19 July 2019, *G. Parolly & al. 15742* (B 10 1118222), PP004941, PP004614, PP004288.

*Campanulacordifolia* K. Koch: CAM1043 [DB 41271]: Russian Federation, N Caucasus, Krasnodar Kra, Novorossiysk district, khrebet Markotkh above Novorossiysk bay, near pereval Andreevskiy (= Sem’ Vetrov), 450-550 m, 37.86667°N, 44.72556°E, 20 July 2015, *E. von Raab-Straube & al. 6730* (B 10 1052507), PP004636, PP004311, PP003985.

*Campanuladamascena* Labill.: CAM1244 [DB 41840]: Palestinian Territory, Wadi Tawahin (Safad), 3 May 1942, *P. H. Davis 4538* (ERE 33388), PP004913, PP004571, PP004245.

*Campanuladasyantha* M. Bieb.: CAM1245 [DB 41841]: Russia, Buryatia, Khentei-Chikoy Highlands, Chikokonskiy Khrebet, “Golets” (mountain summit), along the watershed of the Goronkova and Bystrinsky valleys, *M. Maksimova & A. Maksimova* s.n. - *1967-06-30* (ERE 30106), PP004887, PP004572, PP004246.

*Campanuladaghestanica* Fomin: CAM1150 [DB 41667]: Russian Federation, Dagestan, N Caucasus, Akushinskiy raion, surroundings of village Akusha, slope of Mt. Maara, 1770 m, 47.30439°N, 42.24453°E, 13 June 2016, *R. Murtazaliyev* s.n. (herb. Murtazaliyev), PP004774, PP004449, PP004123.

*Campanuladolomitica* E.A. Busch: CAM1301 [DB 40131]: Russian Federation, N Caucasua, North Ossetia-Alania, Fiagdon valley, 1135 m, 44.32639°N, 42.88167°E, 22 July 2019, *G. Parolly & al. 15800* (B 10 1118226), PP004944, PP004618, PP004292.

*Campanuladoluchanovii* Kharadze: CAM1273 [DB 41866]: Georgia, N Caucasus, Kakheti (Tusheti), Akhmeta district, Chesho village, 2000 m, 21 August 1926, *D. Mtskhvetadze & M. Pataraia* s.n. (TBI 1038259), PP004898, PP004593, PP004266; CAM1329 (Type) [DB 45126]: Georgia, N Caucasus, Lagodekhi reserve, Khochal-dag tract, to the west from marker 9289’ (according 5-mile map), 2700 m, 27 Jul 1938, *A. Doluchanov* s.n. (TBI 1024822), PP004951, PP004625, PP004299.

*Campanulafominii* Grossh.: CAM1246 [DB 41842]: Azerbaijan, N Caucasus, Qusar, At the foot of the Mountain Qızılqaya, 6 August 1952, *A. A. Fedorov* s.n. (ERE 69605), PP004914, PP004573, PP004247; CAM1247 [DB 41843]: Azerbaijan, N Caucasus, Qusar, River Qusar, eastern of village Laza, Mountain Qızılqaya, 2400 m, 26 August 1976, s.n. (ERE 69623), PP004915, PP004574, PP004248; CAM1267 [DB 41860]: Azerbaijan, N Caucasus, At the foot of the Mountain Qızılqaya, 6 August 1952, *A. A. Fedorov* s.n. (TBI 1038314), PP004896, PP004588, PP004262; CAM1268 [DB 41861]: Azerbaijan, N Caucasus, between village Laza and Mountain Shahdagh, along the right side of the valley, 18 July 1966, *A. Kharadze* s.n. (TBI 1035163), PP004897, PP004589, PP004263; CAM1284 [DB 41877]: Azerbaijan, N Caucasus, Laza village, 1850 m, 17 July 1966, *N. Cholokashvili & A. Kharadze* s.n. (TBI 1038315), PP004902, PP004602, PP004276; CAM1285 [DB 41878]: Azerbaijan, N Caucasus, between village Laza and Mountain Shahdagh, along the right side of the valley, 18 July 1966, *A. Kharadze* s.n. (TBI 1035165), PP004930, PP004603, PP004277.

Campanulaaff.glomerata L.: CAM1046 [DB 41274]: *E. von Raab-Straube & al. 6834* (B 10 1052511), PP004638, PP004313, PP003987, from the same population as *6832*; CAM1048 [DB 41276]: *E. von Raab-Straube & al. 6838* (B 10 1052513), PP004639, PP004314, PP003988, from the same population as *6832*; CAM1049 [DB 41277]: *E. von Raab-Straube & al. 6839* (B 10 1052514), PP004640, PP004315, PP003989, from the same population as *6832*; CAM1063 [DB 41290]: *E. von Raab-Straube & al. 6828* (B 10 1052533), PP004646, PP004321, PP003995, from the same population as *6832*; CAM1115 [DB 41503]: Georgia, N Caucasus, Racha-Lechkhumi and Kvemo Svaneti (Racha), Oni district, Shkmeri village, 1920 m, 43.41615°N, 42.5066°E, 21 July 2016, *N. Silakadze & al. 40* (B 10 1052247), PP004696, PP004371, PP004045; CAM1118 [DB 41506]: Georgia, N Caucasus, Racha-Lechkhumi and Kvemo Svaneti (Racha), Oni district, Shkmeri village, 1650 m, 43.40138°N, 42.48907°E, 21 July 2016, *N. Silakadze & al. 42* (B 10 1052250), PP004698, PP004373, PP004047.

*Campanulakadargavanica* Amirkh. & Komzha: CAM546, [DB 16536]: Russian Federation, N Caucasus, North Ossetia-Alania, 24 June 1982, *Amijanov* s.n (LE), *petD* JX915017, *rpl16* PP004301.

*Campanulakryophila* Rupr.: CAM1248 [DB 41844]: Russian Federation, N Caucasus, North Ossetia-Alania, River Ardon, Tsey, 14 July 1983, *Yu. L. Menitsky et al.* s.n. *1983-07-14* (ERE 69613), PP004916, PP004575, PP004249; CAM1279 [DB 41872]: Russian Federation, N Caucasus, North Ossetia-Alania, Bass. R. Ardon, near the glacier Tsey, 9 August 1954, *A. Kharadze & R. Gagnidze* s.n. (TBI 1037313), PP004927, PP004599, PP004272; CAM1281 [DB 41874]: Russian Federation, N Caucasus, North Ossetia-Alania, Glacier Tsey, 5 July 1915, *N. Vvedensky 1309* (TBI 1034882), PP004929, PP004627, PP004274.

*Campanulalactiflora* M. Bieb.: CAM1068 [DB 41297]: Russian Federation, N Caucasus, Republic of Adygea, Apsheronskiy rayon, road to Lagonaki plateau ca. 1.5 km S of Bol’shaya Azishskaya peshchera [= big Azish cave], 1500 m, 40.01944°N, 44.10833°E, 28 July 2015, *E. von Raab-Straube & al. 6909* (B 10 1052539), PP004650, PP004325, PP003999; CAM1139 [DB 41630]: Georgia, S Caucasus, Samckhe-Javakheti, Borjomi district, on the road from Tskhratskaro Pass to Bakuriani, 1820 m, 43.49492°N, 41.72062°E, 10 August 2016, *N. Silakadze 64-2* (B 10 1052314), PP004747, PP004422, PP004096.

*Campanulalatifolia* L.: CAM1069 [DB 41298]: Russian Federation, N Caucasus, Republic of Adygea, Maikopskiy rayon, Lagonaki plateau, just S of lake Psenodakh, 1950-2000 m, 39.9°N, 44.00833°E, 29 July 2015, *E. von Raab-Straube & al. 6926* (B 10 1052540), PP004651, PP004326, PP004000; CAM1114 [DB 41502]: Georgia, N Caucasus, Racha-Lechkhumi and Kvemo Svaneti (Racha), Oni district, Shkmeri village, 1920 m, 43.41615°N, 42.5066°E, 21 July 2016, *N. Silakadze & al. 39* (B 10 1052246), PP004695, PP004370, PP004044.

*Campanulaledebouriana* Trautv.: CAM1265 [DB 41858]: Turkey, S Caucasus, Kars Province, the ridge between the Araks and the Upper Euphrates, 19 July 1886, *W. Massalski* s.n. (TBI 1035047), PP004894, PP004586, PP004260; CAM1282 [DB 41875]: Turkey, S Caucasus, Kars Province, Digor-chay valley, 15 July 1886, *W. Massalski* s.n. (TBI 1035046), PP004901, PP004601, PP004275.

*Campanulameyeriana* Rupr.: CAM1249 [DB 41845]: Azerbaijan, N Caucasus, Qusar, Right side of the river Qusar, Kuzun village, 10 July 1978, *Yu. L. Menitsky & al. 170* (ERE 70847), PP004888, PP004576, PP004250; CAM1269 [DB 41862]: Azerbaijan, N Caucasus, Prov. Baku, distr. Kuba, prope pag. Kryz, 15 July 1929, *M. Sachokia* s.n. (TBI 1035166), PP004921, PP004590, PP004264; CAM1330 (Type) [DB 45127]: Azerbaijan, N Caucasus, between village Laza and Mountain Shahdagh, along the right side of the valley, 18 July 1966, *A. Kharadze* s.n. (TBI 1024863), PP004952, PP004626, PP004300.

*Campanulaminsteriana* Grossh.: CAM1262 [DB 41855]: Armenia, in districtu Daralakhez (in the district of Daralakhez), Meridiem versus a pago Chaczik (facing south from the village of Chaczik), 21 July 1950, *A. Takhtadzhian & S. Czerepanov 4186b* (TBI 1034828), PP004893, PP004585, PP004259; CAM1278 [DB 41871]: Azerbaijan, S Caucasus, Nakhchivan, in monte Karakusch prope pag. Aznabyurt (In the mountains of Karakusch near the village of Aznabyurt), 8 July 1947, *A. Grossheim & al. 4186a* (TBI 1034827), PP004900, PP004598, PP004271.

*Campanulaossetica* M. Bieb.: CAM1300 [DB 40130]: Russian Federation, N Caucasus, North Ossetia-Alania, Fiagdon valley, 1135 m, 44.32638°N, 42.88166°E, 22 July 2019, *G. Parolly & al. 15799* (B 10 1118224), PP004943, PP004617, PP004291.

*Campanulapetrophila* Rupr.: CAM1085a [DB 41376]: Georgia, N Caucasus, Kakheti (Tusheti), Akhmeta district, Abano Pass, 2310 m, 45.51258°N, 42.28558°E, 6 July 2016, *N. Silakadze & al. 8a* (B 10 1052209), PP004664, PP004339, PP004013; CAM1090a [DB 41397]: Georgia, N Caucasus, Kakheti (Tusheti), Akhmeta district, Dano village, 2140 m, 45.5661°N, 42.4508°E, 7 July 2016, *N. Silakadze & al. 13a* (B 10 1052214), PP004668, PP004343, PP004017; CAM1090o [DB 41411]: *N. Silakadze & al. 13o* (B 10 1052215), PP004669, PP004344, PP004018, from the same population as *13a*; CAM1093a [DB 41415]: Georgia, N Caucasus, Kakheti (Tusheti), Akhmeta district, between villages Bochorna and Dochu, 2230 m, 45.55618°N, 42.3945°E, 8 July 2016, *N. Silakadze & al. 18a* (B 10 1052218), PP004672, PP004347, PP004021; CAM1093b [DB 41416]: *N. Silakadze & al. 18b* (B 10 1052219), PP004673, PP004348, PP004022, from the same population as *18a*; CAM1095a [DB 41418]: Georgia, N Caucasus, Kakheti (Tusheti), Akhmeta district, between villages Dochu and Begelta, 2190 m, 45.54946°N, 42.40073°E, 8 July 2016, *N. Silakadze & al. 20a* (B 10 1052221), PP004675, PP004350, PP004024; CAM1095g [DB 41424]: Georgia, N Caucasus, Kakheti (Tusheti), Akhmeta district, between villages Dochu and Begelta, 2020 m, 45.53849°N, 42.40451°E, 8 July 2016, *N. Silakadze & al. 20g* (B 10 1052222), PP004676, PP004351, PP004025; CAM1095k [DB 41428]: Georgia, N Caucasus, Kakheti (Tusheti), Akhmeta district, between villages Dochu and Begelta, 1980 m, 45.53567°N, 42.40676°E, 8 July 2016, *N. Silakadze & al. 20k* (B 10 1052223), PP004677, PP004352, PP004026; CAM1097 [DB 41430]: Georgia, N Caucasus, Kakheti (Tusheti), Akhmeta district, Begelta village, 1980 m, 45.53567°N, 42.40676°E, 8 July 2016, *N. Silakadze & al. 22* (B 10 1052225), PP004679, PP004354, PP004028; CAM1099 [DB 41432]: Georgia, N Caucasus, Kakheti (Tusheti), Akhmeta district, Dartlo village, 1910 m, 45.58929°N, 42.4276°E, 9 July 2016, *N. Silakadze & al. 24* (B 10 1052227), PP004680, PP004355, PP004029; CAM1102 [DB 41435]: Georgia, N Caucasus, Kakheti (Tusheti), Akhmeta district, Dartlo village, 1840 m, 45.55722°N, 42.44881°E, 9 July 2016, *N. Silakadze & al. 27* (B 10 1052230), PP004683, PP004358, PP004032; CAM1103j [DB 41445]: Georgia, N Caucasus, Kakheti (Tusheti), Akhmeta district, road to Chesho village, 1920 m, 45.54597°N, 42.47004°E, 9 July 2016, *N. Silakadze & al. 28j* (B 10 1052231), PP004684, PP004359, PP004033; CAM1103t [DB 41455]: Georgia, N Caucasus, Kakheti (Tusheti), Akhmeta district, road to Chesho village, 1940 m, 45.54396°N, 42.47186°E, 9 July 2016, *N. Silakadze & al. 28t* (B 10 1052232), PP004685, PP004360, PP004034; CAM1105a [DB 41472]: Georgia, N Caucasus, Kakheti (Tusheti), Akhmeta district, Kvavlo village, 2180 m, 45.58342°N, 42.44753°E, 9 July 2016, *N. Silakadze & al. 30a* (B 10 1052234), PP004687, PP004362, PP004036; CAM1154 [DB 41671]: Russian Federation, Dagestan, N Caucasus, Rutulskiy raion, Vug river gorge, below village Djinykh, northern rocky slope, 1810 m, 47.05497°N, 41.66447°E, 26 July 2016, *R. Murtazaliyev* s.n. (herb. Murtazaliyev), PP004775, PP004450, PP004124.

*Campanularadchensis* Kharadze: CAM1111b [DB 41498]: Georgia, N Caucasus, Racha-Lechkhumi and Kvemo Svaneti (Racha), Oni district, Shkmeri village, Kvagakhetkila, 1660 m, 43.39915°N, 42.51237°E, 21 July 2016, *N. Silakadze & al. 36b* (B 10 1052242), PP004692, PP004367, PP004041; CAM1111c [DB 41499]: *N. Silakadze & al. 36c* (B 10 1052243), PP004693, PP004368, PP004042, from the same population as *36b*; CAM1206b [DB 41774]: Georgia, N Caucasus, Racha-Lechkhumi and Kvemo Svaneti (Racha), Oni district, Shkmeri village, Kvagakhetkila, 1660 m, 43.3993°N, 42.51266°E, 5 July 2017, *N. Silakadze & al. 107b* (B 10 1052436), PP004849, PP004525, PP004199; CAM1207a [DB 41775]: Georgia, N Caucasus, Racha-Lechkhumi and Kvemo Svaneti (Racha), Ambrolauri district, Zemo Tlughi village, Mountain Satsalike, 1900 m, 43.19407°N, 42.40336°E, 6 July 2017, *N. Silakadze & al. 108a* (B 10 1052437), PP004850, PP004526, PP004200.

*Campanularapunculoides* L.: CAM1032 [DB 41209]: Russian Federation, N Caucasus, Stavropol Krai, Pyatigorsk, NE slopes of Mt. Mashuk, 620 m, 43.11139°N, 44.05389°E, 25 June 2015, *T. Borsch & al. 5779* (B 10 1052484), PP004628, PP004303, PP003977; CAM1053 [DB 41280]: Russian Federation, N Caucasus, Krasnodar Krai, Mostovskiy rayon, Skalistyy khrebet, NW of Psebay, khrebet Gerpegem, 1100 m, 40.77778°N, 44.13972°E, 27 July 2015, *E. von Raab-Straube & al. 6867* (B 10 1052518), PP004643, PP004318, PP003992; CAM1061 [DB 41288]: Russian Federation, N Caucasus, Krasnodar Krai, Mostovskiy rayon, Skalistyy khrebet, Shakhan mountain between Malaya Laba and Bol’shaya Laba ca. 4 km E of Andryuki, 1150 m, 40.88833°N, 44.105°E, 25 July 2015, *E. von Raab-Straube & al. 6831* (B 10 1052531), PP004644, PP004319, PP003993; CAM1066 [DB 41293]: Russian Federation, N Caucasus, Krasnodar Krai, Apsheronskiy rayon, ca. 1 km S of Mezmay, verkhneye Kurdzhipskoye ushchel’ye [= gorge of upper Kurdzhips river], 700 m, 39.96306°N, 44.18861°E, 28 July 2015, *E. von Raab-Straube & al. 6903* (B 10 1052537), PP004648, PP004323, PP003997; CAM1113 [DB 41501]: Georgia, N Caucasus, Racha-Lechkhumi and Kvemo Svaneti (Racha), Oni district, Shkmeri village, 1920 m, 43.41615°N, 42.5066°E, 21 July 2016, *N. Silakadze & al. 38* (B 10 1052245), PP004694, PP004369, PP004043; CAM1119 [DB 41507]: Georgia, N Caucasus, Racha-Lechkhumi and Kvemo Svaneti (Racha), Oni district, Shkmeri village, 1850 m, 43.343716°N, 42.51512°E, 21 July 2016, *N. Silakadze & al. 43* (B 10 1052251), PP004699, PP004374, PP004048.

*Campanularuprechtii* Boiss.: CAM1255 [DB 41851]: Armenia, S Caucasus, Kapan region, Kaputjugh Mountain, 22 August 1989, *M. Oganesian* s.n. (ERE 118764), PP004918, PP004581, PP004255; CAM1257 [DB 41853]: Armenia, S Caucasus, Syunik province, Near lake Tsakhkar, 3100 m, 46.074°N, 39.061°E, 21 July 2008, *M. Aghabalyan & A. Malkhasian* s.n. (ERE 182056), PP004919, PP004582, PP004256; CAM1276 [DB 41869]: Armenia, S Caucasus, Zangezur, Bargushat range, 3000 m, 1 August 1951, *Akhverdov & Mirzoeva* s.n. (TBI 1036558), PP004925, PP004596, PP004269; CAM1293 [DB 41886]: Armenia, S Caucasus, Zangezur, Bargushat range, 3000 m, 1 August 1951, *Akhverdov* s.n. (TBI 1036645), PP004937, PP004610, PP004284; CAM1294 [DB 41887]: Armenia, S Caucasus, East spur of Mountain Soyukh, (Soyugdagh Mountain), 2500 m, 7 June 1947, *A. A. DoluChanov* s.n. (TBI 1036647), PP004938, PP004611, PP004285; CAM1295 [DB 41888]: Azerbaijan, S Caucasus, Nakhchivan, Peak of Soyukh (Soyugdagh Mountain), 3100 m, 9 August 1968, *D. Mtskhvetadze & D. Kapanadze* s.n. (TBI 1036648), PP004939, PP004612, PP004286.

*Campanulasaxifraga* M. Bieb.: CAM1122a [DB 41510]: Georgia, N Caucasus, Racha-Lechkhumi and Kvemo Svaneti (Lechkhumi), Tsageri district, Khvamli Mountain, 1920 m, 42.71693°N, 42.50947°E, 22 July 2016, *N. Silakadze & al. 46a* (B 10 1052254), PP004701, PP004376, PP004050; CAM1122l [DB 41521]: *N. Silakadze & al. 46l* (B 10 1052255), PP004702, PP004377, PP004051, from the same population as *46a*; CAM1122m [DB 41522]: *N. Silakadze & al. 46m* (B 10 1052256), PP004703, PP004378, PP004052, from the same population as *46a*; CAM1122n [DB 41523]: *N. Silakadze & al. 46n* (B 10 1052257), PP004704, PP004379, PP004053, from the same population as *46a*; CAM1124a [DB 41538]: Georgia, N Caucasus, Racha-Lechkhumi and Kvemo Svaneti (Lechkhumi), Tsageri district, Askhi Mountain, 1930 m, 42.5564°N, 42.6281°E, 23 July 2016, *N. Silakadze & al. 49a* (B 10 1052259), PP004705, PP004380, PP004054; CAM1124b [DB 41539]: *N. Silakadze & al. 49b* (B 10 1052260), PP004706, PP004381, PP004055, from the same population as *49a*; CAM1124c [DB 41540]: *N. Silakadze & al. 49c* (B 10 1052261), PP004707, PP004382, PP004056, from the same population as *49a*; CAM1124d [DB 41541]: *N. Silakadze & al. 49d* (B 10 1052262), PP004708, PP004383, PP004057, from the same population as *49a*; CAM1124e [DB 41542]: *N. Silakadze & al. 49e* (B 10 1052263), PP004709, PP004384, PP004058, from the same population as *49a*; CAM1124f [DB 41543]: *N. Silakadze & al. 49f* (B 10 1052264), PP004710, PP004385, PP004059, from the same population as *49a*; CAM1127a [DB 41546]: Georgia, N Caucasus, Samegrelo-Zemo Svaneti (Samegrelo), Martvili district, Taleri village, Chegola Mountain, 2500 m, 42.42575°N, 42.77593°E, 26 July 2016, *N. Silakadze & al. 52a* (B 10 1052267), PP004712, PP004387, PP004061; CAM1127b [DB 41547]: *N. Silakadze & al. 52b* (B 10 1052268), PP004713, PP004388, PP004062, from the same population as *52a*; CAM1127c [DB 41548]: *N. Silakadze & al. 52c* (B 10 1052269), PP004714, PP004389, PP004063, from the same population as *52a*; CAM1127d [DB 41549]: *N. Silakadze & al. 52d* (B 10 1052270), PP004715, PP004390, PP004064, from the same population as *52a*; CAM1127e [DB 41550]: *N. Silakadze & al. 52e* (B 10 1052271), PP004716, PP004391, PP004065, from the same population as *52a*; CAM1127f [DB 41551]: *N. Silakadze & al. 52f* (B 10 1052272), PP004717, PP004392, PP004066, from the same population as *52a*; CAM1127g [DB 41552]: *N. Silakadze & al. 52g* (B 10 1052273), PP004718, PP004393, PP004067, from the same population as *52a*; CAM1130g [DB 41572]: Georgia, N Caucasus, Samegrelo-Zemo Svaneti (Zemo Svaneti), Mestia district, Mestia-Koruldi Lake, 2630 m, 42.7113°N, 43.08155°E, 28 July 2016, *N. Silakadze & al. 55g* (B 10 1052282), PP004722, PP004397, PP004071; CAM1130j [DB 41575]: *N. Silakadze & al. 55j* (B 10 1052285), PP004723, PP004398, PP004072, from the same population as *55g*; CAM1130l [DB 41577]: *N. Silakadze & al. 55l* (B 10 1052287), PP004725, PP004400, PP004074, from the same population as *55g*; CAM1130m [DB 41578]: *N. Silakadze & al. 55m* (B 10 1052288), PP004726, PP004401, PP004075, from the same population as *55g*; CAM1130n [DB 41579]: Georgia, N Caucasus, Samegrelo-Zemo Svaneti (Zemo Svaneti), Mestia district, Mestia-Koruldi Lake, 2690 m, 42.70922°N, 43.08383°E, 28 July 2016, *N. Silakadze & al. 55n* (B 10 1052289), PP004727, PP004402, PP004076; CAM1130p [DB 41581]: Georgia, N Caucasus, Samegrelo-Zemo Svaneti (Zemo Svaneti), Mestia district, Mestia-Koruldi Lake, 2720 m, 42.7081°N, 43.08475°E, 28 July 2016, *N. Silakadze & al. 55p* (B 10 1052291), PP004728, PP004403, PP004077; CAM1208a [DB 41778]: Georgia, N Caucasus, Racha-Lechkhumi and Kvemo Svaneti (Lechkhumi), Tsageri district, Khvamli Mountain, 1910 m, 42.71736°N, 42.50833°E, 8 July 2017, *N. Silakadze & al. 109a* (B 10 1052440), PP004851, PP004527, PP004201; CAM1208b [DB 41779]: *N. Silakadze & al. 109b* (B 10 1052441), PP004852, PP004528, PP004202, from the same population as *109a*; CAM1208c [DB 41780]: *N. Silakadze & al. 109c* (B 10 1052442), PP004853, PP004529, PP004203, from the same population as *109a*; CAM1208d [DB 41781]: *N. Silakadze & al. 109d* (B 10 1052443), PP004854, PP004530, PP004204, from the same population as *109a*; CAM1210b [DB 41784]: Georgia, N Caucasus, Racha-Lechkhumi and Kvemo Svaneti (Lechkhumi), Tsageri district, Zemo Lukhvano village, Tsekuri Mountain, 2270 m, 42.62859°N, 42.69225°E, 12 July 2017, *N. Silakadze & al. 111b* (B 10 1052446), PP004855, PP004531, PP004205; CAM1210c [DB 41785]: *N. Silakadze & al. 111c* (B 10 1052447), PP004856, PP004532, PP004206, from the same population as *111b*; CAM1212a [DB 41787]: Georgia, N Caucasus, Racha-Lechkhumi and Kvemo Svaneti (Kvemo Svaneti), Lentekhi district, between Rhobi and Zeskho villages, 1530 m, 43.16454°N, 42.85818°E, 13 July 2017, *N. Silakadze & al. 113a* (B 10 1052449), PP004857, PP004533, PP004207; CAM1212b [DB 41788]: *N. Silakadze & al. 113b* (B 10 1052450), PP004858, PP004534, PP004208, from the same population as *113a*; CAM1214a [DB 41790]: Georgia, N Caucasus, Samegrelo-Zemo Svaneti (Zemo Svaneti), Ushguli village, Shkhara Glacier, 2510 m, 43.08811°N, 42.96178°E, 15 July 2017, *N. Silakadze & al. 115a* (B 10 1052452), PP004859, PP004535, PP004209; CAM1214b [DB 41791]: *N. Silakadze & al. 115b* (B 10 1052453), PP004860, PP004536, PP004210, from the same population as *115a*; CAM1214c [DB 41792]: Georgia, N Caucasus, Samegrelo-Zemo Svaneti (Zemo Svaneti), Ushguli village, Shkhara Glacier, 2470 m, 43.08767°N, 42.9605°E, 15 July 2017, *N. Silakadze & al. 115c* (B 10 1052454), PP004861, PP004537, PP004211; CAM1215a [DB 41794]: Georgia, N Caucasus, Racha-Lechkhumi and Kvemo Svaneti (Kvemo Svaneti), Lentekhi district, Zeskho village, Shavi Utsnobi Mountain, 2120 m, 43.21659°N, 42.90786°E, 16 July 2017, *N. Silakadze & al. 116a* (B 10 1052456), PP004862, PP004538, PP004212; CAM1215b [DB 41795]: *N. Silakadze & al. 116b* (B 10 1052457), PP004863, PP004539, PP004213, from the same population as *116a*; CAM1215c [DB 41796]: *N. Silakadze & al. 116c* (B 10 1052458), PP004864, PP004540, PP004214, from the same population as *116a*; CAM1216a [DB 41797]: Georgia, N Caucasus, Racha-Lechkhumi and Kvemo Svaneti (Kvemo Svaneti), Lentekhi district, Koruldashi village, Ailama Mountain, 2390 m, 43.15861°N, 42.92956°E, 17 July 2017, *N. Silakadze & al. 117a* (B 10 1052459), PP004865, PP004541, PP004215; CAM1216b [DB 41798]: *N. Silakadze & al. 117b* (B 10 1052460), PP004866, PP004542, PP004216, from the same population as *117a*; CAM1216c [DB 41799]: *N. Silakadze & al. 117c* (B 10 1052461), PP004867, PP004543, PP004217, from the same population as *117a*; CAM1216d [DB 41800]: Georgia, N Caucasus, Racha-Lechkhumi and Kvemo Svaneti (Kvemo Svaneti), Lentekhi district, Koruldashi village, Ailama Mountain, 2430 m, 43.15974°N, 42.93037°E, 17 July 2017, *N. Silakadze & al. 117d* (B 10 1052462), PP004868, PP004544, PP004218; CAM1216f [DB 41802]: *N. Silakadze & al. 117f* (B 10 1052464), PP004869, PP004545, PP004219, from the same population as *117d*; CAM1216g [DB 41803]: *N. Silakadze & al. 117g* (B 10 1052465), PP004870, PP004546, PP004220, from the same population as *117d*; CAM1216h [DB 41804]: *N. Silakadze & al. 117h* (B 10 1052466 PP004871, PP004547, PP004221, from the same population as *117d*; CAM1237 [DB 41833]: Russian Federation, N Caucasus, Kabardino-Balkaria, North side of Elbruzi Mountain, Massive of the river Malka, 29 July 1981, *Yu. L. Menitsky et al. 430* (ERE 70768), PP004912, PP004565, PP004239; CAM1250 [DB 41846]: Russian Federation, N Caucasus, Karachay-Cherkessia, Top of the Bol’shaya Laba river, on the hill of Zakan, 8 July 1979 *T. Zaikonnikova et al.* s.n. (ERE 70757), PP004889, PP004577, PP004251; CAM1251 [DB 41847]: Russian Federation, N Caucasus, Karachay-Cherkessia, Karachayevsky raion, the right side of river Kuban, village Khurzuk, 1600-1800 m, 7 August 1989, *N. Khandilyan* s.n. (ERE 70759), PP004890, PP004578, PP004252; CAM1270 [DB 41863]: Georgia, N Caucasus, Samegrelo-Zemo Svaneti (Zemo Svaneti), Gorges of Tsaneri, Khozheri, 2800 m, 1 September *B. Zurebiani* s.n. (TBI 1038399), PP004922, PP004591, PP004265; CAM1277 [DB 41870]: Russian Federation, N Caucasus, Karachay-Cherkessia, Uchkulan village, right side of river Kuban, 14 August 1956, *A. Kharadze et al.* s.n. (TBI 1033287), PP004926, PP004597, PP004270; CAM1290 [DB 41883]: Georgia, N Caucasus, Racha-Lechkhumi and Kvemo Svaneti (Racha), Ghebi village, 2400- 2600 m, 5 August 1982, *Sh. Shetekauri* s.n. (TBI 1037294), PP004934, PP004607, PP004281; CAM1312 [DB 41891]: Russian Federation, N Caucasus, Kabardino-Balkaria, Elbrus Mountain, 3800 m, September 1980, *W. Heller* s.n. (B 10 0507479), PP004950, PP004624, PP004298.

Campanulaaff.sibirica L.: CAM1036 [DB 41213]: Russian Federation, N Caucasus, Stavropol Kray, Predgorny rayon, W side of Podkumok river valley ca. 8 km N of Kislovodsk on road (A157) to Yessentuki, 670-700 m, 42.79417°N, 43.99583°E, 25 June 2015, *T. Borsch & al. 5810* (B 10 1052494), PP004629, PP004304, PP003978; CAM1045 [DB 41273]: Russian Federation, N Caucasus, Krasnodar Krai, Mostovskiy rayon, Skalistyy khrebet, Shakhan mountain between Malaya Laba and Bol’shaya Laba ca. 4 km E of Andryuki, 1150 m, 40.88833°N, 44.105°E, 25 July 2015, *E. von Raab-Straube & al. 6832* (B 10 1052510), PP004637, PP004312, PP003986; CAM1050 [DB 41278]: *E. von Raab-Straube & al. 6840* (B 10 1052515), PP004641, PP004316, PP003990, from the same population as *6832*; CAM1051 [DB 41279]: *E. von Raab-Straube & al. 6841* (B 10 1052516), PP004642, PP004317, PP003991, from the same population as *6832*; CAM1064 [DB 41291]: *E. von Raab-Straube & al. 6827* (B 10 1052534), PP004647, PP004322, PP003996, from the same population as *6832*.

Campanulaaff.sibiricasubsp.hohenackeri (Fisch. & C. A. Mey.) Damboldt: CAM1091a [DB 41412]: Georgia, N Caucasus, Kakheti (Tusheti), Akhmeta district, Dano village, 2140 m, 45.5661°N, 42.4508°E, 7 July 2016, *N. Silakadze & al. 15a* (B 10 1052216), PP004670, PP004345, PP004019; CAM1096 [DB 41429]: Georgia, N Caucasus, Kakheti (Tusheti), Akhmeta district, Begelta village, 1980 m, 45.53567°N, 42.40676°E, 8 July 2016, *N. Silakadze & al. 21* (B 10 1052224), PP004678, PP004353, PP004027; CAM1161 [DB 41678]: Russian Federation, Dagestan, N Caucasus, Levashinskiy raion, surroundngs of village Tsudarakh, 1250 m, 47.16505°N, 42.32419°E, 13 July 2016, *R. Murtazaliyev* s.n. (herb. Murtazaliyev), PP004776, PP004451, PP004125; CAM1162 [DB 41679]: Russian Federation, Dagestan, N Caucasus, Dakhadayevsky raion, Urkarakh - Kubachi, on rocky places along the port side at the bridge over the river Khulakherk, 910 m, 47.61794°N, 42.12358°E, 28 July 2016, *R. Murtazaliyev* s.n. (herb. Murtazaliyev), PP004777, PP004452, PP004126; CAM1260 [DB 27374]: Georgia, N Caucasus, Kakheti, Akhmeta district, Tusheti, Tusheti Protected Area, Upper Omalo road, below Omalo Castle, 2040 m, 8 July 2016, *G. Parolly & al. 15045* (B 10 0518297 PP004892, PP004583, PP004257.

*Campanulastevenii* M. Bieb.: CAM1087a [DB 41382]: Georgia, N Caucasus, Kakheti (Tusheti), Akhmeta district, Dano village, 2150 m, 45.56757°N, 42.45046°E, 7 July 2016, *N. Silakadze & al. 10a* (B 10 1052211), PP004665, PP004340, PP004014; CAM1193 [DB 41741]: Georgia, S Caucasus, Samckhe-Javakheti, On the way to Akhalkalaki Diago village, 1940 m, 43.49905°N, 41.60252°E, 16 July 2017, *N. Silakadze & K. E. Jones 94* (B 10 1052403), PP004821, PP004497, PP004171.

*Campanulasosnowskyi* Kharadze: CAM1275 [DB 41868]: Russian Federation, N Caucasus, North Ossetia-Alania, Environs of village Chmi, left side of Didneua-kom gorge, 13 July 1947, *A. Kharadze & K. Khutsishvili* s.n. (TBI 1036039), PP004924, PP004595, PP004268; CAM1306 [DB 40158]: Russian Federation, N Caucasua, North Ossetia-Alania, Alagir district, middle course of the Fiagdon river, Kadargavan canyon, 1085 m, 44.32583°N, 42.88111°E, 28 July 2013, *D. S. Shilnikov*, s.n. (*D. S. Shilnikov* n.s), PP004947, PP004621, PP004295.

*Campanulatridentata* Schreb.: CAM1134a [DB 41620]: Georgia, S Caucasus, Samckhe-Javakheti, Borjomi district, Bakuriani, Tskhratskaro Pass, 2460 m, 43.51987°N, 41.68785°E, 10 August 2016, *N. Silakadze 61a* (B 10 1052305), PP004738, PP004413, PP004087; CAM1134b [DB 41621]: *N. Silakadze 61b*, PP004739, PP004414, PP004088, from the same population as *61a*; CAM1140a [DB 41631]: Georgia, S Caucasus, Samckhe-Javakheti, Borjomi district, Tsikhisjvari village, Kodiani Mountain, 2390 m, 43.36105°N, 41.72778°E, 11 August 2016, *N. Silakadze 65a* (B 10 1052315), PP004748, PP004423, PP004097; CAM1140c [DB 41633]: Georgia, S Caucasus, Samckhe-Javakheti, Borjomi district, Tsikhisjvari village, Kodiani Mountain, 2410 m, 43.36113°N, 41.72723°E, 11 August 2016, *N. Silakadze 65c* (B 10 1052317), PP004749, PP004424, PP004098; CAM1220a [DB 41813]: Armenia, S Caucasus, Aragatsotn Province, Aragats Mountain, surroundings of the Kari lake, 3210 m, 44.17857°N, 40.47334°E, 21 August 2017, *N. Silakadze & al. 121a* (B 10 1052475), PP004877, PP004554, PP004228.

## Supplementary materials

Supplementary material 1 Bayesian 50% majority-rule consensus tree of the combined dataset of the Caucasian *Campanula* species based on three plastid markers (*trnK*/*matK*, *petD* and *rpl16*) Data type pdf https://binary.pensoft.net/file/1079151This dataset is made available under the Open Database License (http://opendatacommons.org/licenses/odbl/1.0/). The Open Database License (ODbL) is a license agreement intended to allow users to freely share, modify, and use this Dataset while maintaining this same freedom for others, provided that the original source and author(s) are credited.Nana Silakadze, Marine Mosulishvili, Thomas Borsch, Norbert Kilian

Supplementary material 2 Maximum parsimony tree of the combined dataset of the Caucasian *Campanula* species based on three plastid markers (*trnK*/*matK*, *petD* and *rpl16*) Data type pdf https://binary.pensoft.net/file/1079152This dataset is made available under the Open Database License (http://opendatacommons.org/licenses/odbl/1.0/). The Open Database License (ODbL) is a license agreement intended to allow users to freely share, modify, and use this Dataset while maintaining this same freedom for others, provided that the original source and author(s) are credited.Nana Silakadze, Marine Mosulishvili, Thomas Borsch, Norbert Kilian

Supplementary material 3 Taxonomy of the *Scapiflorae* group by the different authors Data type docx https://binary.pensoft.net/file/1079153This dataset is made available under the Open Database License (http://opendatacommons.org/licenses/odbl/1.0/). The Open Database License (ODbL) is a license agreement intended to allow users to freely share, modify, and use this Dataset while maintaining this same freedom for others, provided that the original source and author(s) are credited.Nana Silakadze, Marine Mosulishvili, Thomas Borsch, Norbert Kilian

Supplementary material 4 Primers are used for amplification and sequencing reactions Data type docx https://binary.pensoft.net/file/1079154This dataset is made available under the Open Database License (http://opendatacommons.org/licenses/odbl/1.0/). The Open Database License (ODbL) is a license agreement intended to allow users to freely share, modify, and use this Dataset while maintaining this same freedom for others, provided that the original source and author(s) are credited.Nana Silakadze, Marine Mosulishvili, Thomas Borsch, Norbert Kilian
