# Supplementary figures and images for: ﻿The polyphyletic Caucasus-centred Campanula subg. Scapiflorae (Campanulaceae) revisited with a newly circumscribed C. sect. Tridentatae for its core clade
Source: PhytoKeys. 2024 Jun 25;243:149–84. doi: 10.3897/phytokeys.243.120908 (PMC11220400; doi:10.3897/phytokeys.243.120908)

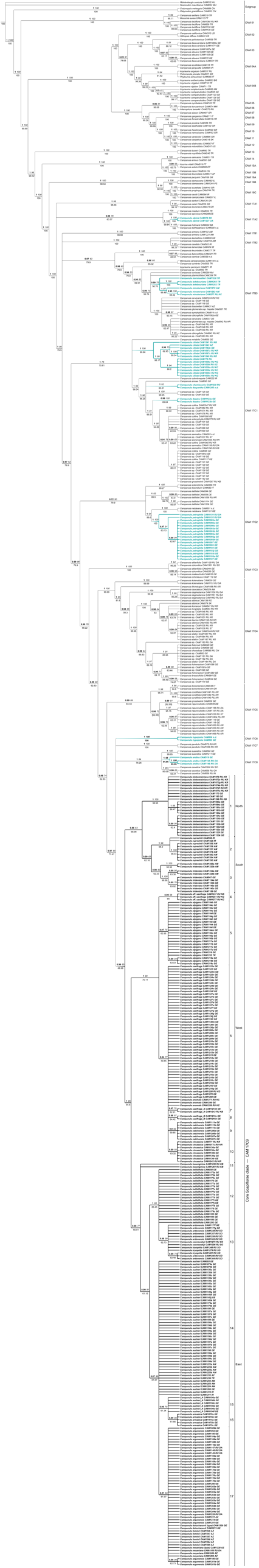

Supplement: Supplementary material 1 — Bayesian 50% majority-rule consensus tree of the combined dataset of the Caucasian Campanula species based on three plastid markers (trnK/matK, petD and rpl16) [file phytokeys-243-149_article-120908__-s001.pdf]

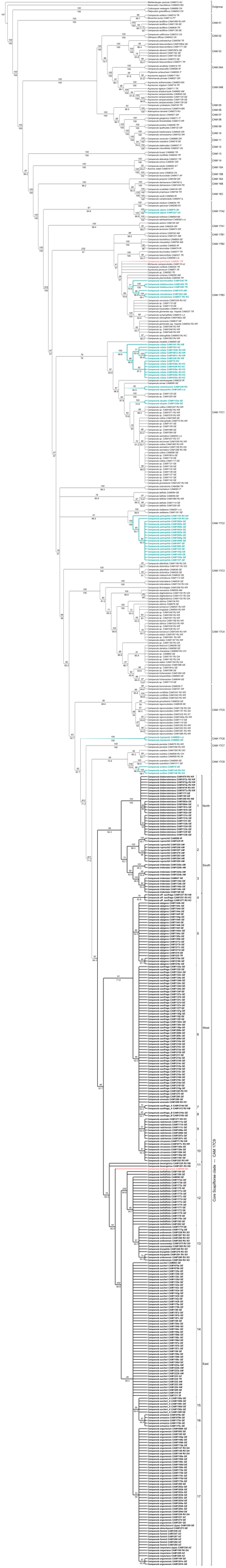

Supplement: Supplementary material 2 — Maximum parsimony tree of the combined dataset of the Caucasian Campanula species based on three plastid markers (trnK/matK, petD and rpl16) [file phytokeys-243-149_article-120908__-s002.pdf]
