# Supplementary material for: ﻿The polyphyletic Caucasus-centred Campanula subg. Scapiflorae (Campanulaceae) revisited with a newly circumscribed C. sect. Tridentatae for its core clade
Source: PhytoKeys. 2024 Jun 25;243:149–84. doi: 10.3897/phytokeys.243.120908 (PMC11220400; doi:10.3897/phytokeys.243.120908)
Supplement: Supplementary material 3 — Taxonomy of the Scapiflorae group by the different authors [file phytokeys-243-149_article-120908__-s003.docx]

**Table S1.** Taxonomy of the *Scapiflorae* group by the different authors. “Taxon names” names in bold indicate taxa preliminarily accepted and names in grey taxa considered as synonyms by the present authors based on the results of this study, exclamation marks indicate that samples were included in the present phylogenetic study, asterisks denote names belonging to *Campanula* sect. *Tridentatae*. In the following columns names in black (not in bold) indicate taxa accepted and names in grey and square brackets indicate names treated as synonyms by the respective authors.

| **Taxon names** | | **Ruprecht (1867)** | **Boissier (1875)** | **Fomin (1905)** | **Kharadze (1949)** |
| --- | --- | --- | --- | --- | --- |
|  |  | ***Campanula* sect. *Medium*** | ***Campanula* ser. *Scapiflorae*** | ***Campanula* ser. *Scapiflorae*** | ***Campanula* sect. *Scapiflorae*** |
|  |  |  |  |  | **(1) Ser. *Ciliatae* Kharadze** |
| 1 | ***C. ciliata* Steven !** | *C. ciliata* | *C. ciliata* | *C. ciliata* | *C. ciliata* |
|  |  |  |  |  |  |
| 2 | ***C. dzaaku* Albov !** |  |  | *C. dzaaku* | *C. dzaaku* |
|  |  |  |  |  | **(2) Ser. *Tridentatae* Kharadze** |
| 3 | ***C. tridentata* Schreb. !*** | *C. tridentata* | *C. tridentata* | *C. tridentata* | *C. tridentata* |
|  | = *C. bithynica* A. DC. * |  |  | [*C. tridentata*] |  |
| 4 | ***C. biebersteiniana* Roem. & Schult. !***  ≡ *C. rupestris* M. Bieb., nom. illeg. * | *C. rupestris* | [*C. tridentata*] | [*C. tridentata*] |  |
|  | = *C. tridens* Rupr. * | *C. tridens* | [*C. tridentata*] | [*C. tridentata*] | *C. tridens* |
|  | = *C. akuschensis* Husseinov* |  |  |  |  |
|  | = *C. scapifoliosa* A. P. Khokhr. * |  |  |  |  |
|  |  |  |  |  | **(3) Ser. *Aucherianae* Kharadze [“*Aucheri*”]** |
| 5 | ***C. aucheri* A. DC. !*** |  | *C. aucheri* | *C. aucheri* | *C. aucheri* |
|  | = *C. froedinii* Rech. f. * |  |  |  |  |
|  | = *C. pallidiflora* Rupr. * |  |  |  |  |
|  | = *C. pubiflora* Rupr. * | *C. pubiflora* | [*C. aucheri*] | [*C. aucheri*] |  |
|  | = *C. hygrophila* Rupr. * | *C. hygrophyla* | [*C. aucheri*] | [*C. aucheri*] |  |
| 6 | ***C. radchensis* Kharadze !*** |  |  |  | *C. radchensis* |
| 7 | ***C. armazica* Kharadze !*** |  |  |  | *C. armazica* |
| 8 | ***C. alpigena* K. Koch !*** |  | [*C. aucheri*] | [*C. aucheri*] | *C. alpigena* |
|  | = *C. fallax* Rupr. * |  | [*C. aucheri*] | [*C. aucheri*] |  |
|  |  |  |  |  |  |
| 9 | ***C. saxifraga* M. Bieb. !*** | *C. saxifraga* | *C. saxifraga* | *C. saxifraga* | *C. saxifraga* |
| 10 | ***C. ruprechtii* Boiss. !*** |  | *C. ruprechtii* |  | *C. ruprechtii* |
|  |  |  |  |  |  |
| 11 | ***C. argunensis* Rupr. !*** | *C. argunensis* | [*C. aucheri*] | *C. argunensis* | *C. argunensis* |
|  | = *C. doluchanovii* Kharadze !* |  |  |  | *C. doluchanovii* |
|  | = *C. meyeriana* Rupr. !* | *C. meyeriana* | [*C. aucheri*] |  |  |
|  |  |  |  |  |  |
| 12 | ***C. bellidifolia* Adams !*** | *C. bellidifolia* | *C. bellidifolia* | *C. bellidifolia* | *C. bellidifolia* |
|  | = *C. adami* M. Bieb. * |  |  | [*C. bellidifolia*] |  |
|  | = *C. sosnowskyi* Kharadze !* |  |  |  | *C. sosnowskyi* |
|  | = *C. fominii* Grossh. !* |  |  |  | *C. fominii* |
| 13 | ***C. czerepanovii* Fed.** |  |  |  |  |
|  |  |  |  |  |  |
|  | = *C. anomala* Fomin !* |  |  | *C. anomala* | *C. anomala* |
|  | = *C. circassica* Fomin !* |  |  | *C. circassica* | *C. circassica* |
|  |  |  |  |  | **(4) Ser. *kryophilae* Kharadze** |
|  | = *C. ardonensis* Rupr. !* | *C. ardonensis* | *C. ardonensis* | *C. ardonensis* | *C. ardonensis* |
|  | = *C. kryophila* Rupr. !* | *C. kryophila* | [*C. ardonensis*] | *C. kryophila* | *C. kryophila* |

**Table S1.** Continued.

| **Taxon names** | | **Ruprecht (1867)** | **Boissier (1875)** | **Fomin (1905)** | **Kharadze (1949)** |
| --- | --- | --- | --- | --- | --- |
|  |  | ***Campanula* sect. *Medium*** | ***Campanula* ser. *Scapiflorae*** | ***Campanula* ser. *Scapiflorae*** | ***Campanula* sect. *Scapiflorae*** |
|  |  |  |  |  |  |
| 14 | ***C. besenginica* Fomin !*** |  |  | *C. besenginica* | *C. besenginica* |
|  |  |  |  |  |  |
| 15 | ***C. dasyantha* M. Bieb. !** |  |  |  |  |
|  | = *C. pallasiana* Roem. & Schult. |  |  |  |  |
|  | = *C. pilosa* Pall. ex Roem. & Schult. |  |  |  |  |
|  | = *C. altaica* A. DC. non Ledeb. |  |  |  |  |
| 16 | ***C. chamissonis* Fed. ex Toyok. & Nosaka !** |  |  |  |  |
|  | = *C. aldanensis* Fed. & Karav. |  |  |  |  |
|  |  |  |  |  |  |
| 17 | ***C. ledebouriana* Trautv. !** |  | *C. ledebourii* |  |  |
| 18 | ***C. petrophila* Rupr. !** | *C. petrophila* |  |  |  |
|  | = *C. zeyensi*s Amirkh. & Tavasiev * |  |  |  |  |
|  | = *C. kadargavanica* Amirkh. & Komzha !* |  |  |  |  |
|  | = *C. songutica* Amirkh. * |  |  |  |  |
| 19 | ***C. bornmuelleri* Nábělek !** |  |  |  |  |
| 20 | ***C. pulvinaris* Hausskn. & Bornm. !** |  |  |  |  |
| 21 | ***C. andina* Rupr. !** |  |  |  |  |
|  | = *C. gumbetica* Boiss. |  |  |  |  |
|  |  |  |  |  |  |
| 22 | ***C. lehmanniana*Bunge** |  |  |  |  |
| 23 | ***C. capusii* (Franch.) Fed.** |  |  |  |  |
|  |  |  |  |  |  |
| 24 | ***C. alpina* Jacq. !** |  |  |  |  |
| 25 | ***C.* *orbelica* Pančić** |  |  |  |  |
|  |  |  |  |  |  |
| 26 | ***C. hypopolia* Trautv. !** |  |  |  |  |
|  |  |  |  |  |  |
| 27 | ***C. minsteriana* Grossh. !** |  |  |  |  |
|  | = *C. karakuschensis* Grossh. |  |  |  |  |
|  | **Species** | **13** | **8** | **12** | **20** |
|  | Subspecies | 0 | 0 | 0 | 0 |
|  | Synonyms | 0 | **9** | **8** | 0 |

**Table S1.** Continued.

| **Taxon names** | | **Fedorov (1957)** | **Kharadze (1976)** | **Oganesian (2000)** | **Victorov (2002)** |
| --- | --- | --- | --- | --- | --- |
|  |  | ***Campanula* subsect. *Scapiflorae*** | ***Campanula* sect. *Scapiflorae*** | ***Campanula* subg. *Scapiflorae*** | ***Campanula* subg. *Scapiflorae*** |
|  |  | **(1) Ser. *Ciliatae* Kharadze** | **(1) Ser. *Ciliatae* Kharadze** |  | **(1) Sect. *Scapiflorae* (Boiss) Kharadze** |
| 1 | ***C. ciliata* Steven !** | *C. ciliata* | *C. ciliata* | *C. ciliata* | *C. ciliata* |
|  |  |  |  |  | **(2) Sect. *Preudocampanula* (Kolak.) Victorov** |
| 2 | ***C. dzaaku* Albov !** | *C. dzaaku* | *C. dzaaku* |  | *C. dzaaku* |
|  |  | **(2) Ser. *Tridentatae* Kharadze** | **(2) Ser. *Tridentatae* Kharadze** |  | **(3) Sect. *Tridentatae*** **(Kharadze) Victorov** |
| 3 | ***C. tridentata* Schreb. !*** | *C. tridentata* | *C. tridentata* | *C. tridentata* subsp. *tridentata* | *C. tridentata* |
|  | = *C. bithynica* A. DC. * |  |  | [*C. tridentata* subsp. *tridentata*] | [*C. tridentata*] |
| 4 | ***C. biebersteiniana* Roem. & Schult. !***  ≡ *C. rupestris* M. Bieb., nom. illeg. * | *C. biebersteiniana* | *C. biebersteiniana* | *C. tridentata* subsp. *biebersteiniana* | [*C. tridentata*] |
|  | = *C. tridens* Rupr. * | [*C. biebersteiniana*] |  | [*C. tridentata* subsp. *biebersteiniana*] | [*C. tridentata*] |
|  | = *C. akuschensis* Husseinov * |  |  | [*C. tridentata* subsp. *biebersteiniana*] | [*C. tridentata*] |
|  | = *C. scapifoliosa* A. P. Khokhr. * |  |  |  | [*C. tridentata*] |
|  |  | **(3) Ser. *Aucherianae* Kharadze** | **(3) Ser. *Aucherianae* Kharadze** |  |  |
| 5 | ***C. aucheri* A. DC. !*** | *C. aucheri* | *C. aucheri* | *C. saxifraga* subsp. *aucheri* | *C. bellidifolia* subsp. *aucheri* |
|  | = *C. froedinii* Rech. f. * |  |  | [*C. saxifraga* subsp. *aucheri*] |  |
|  | = *C. pallidiflora* Rupr. * | [*C. aucheri*] |  |  |  |
|  | = *C. pubiflora* Rupr. * |  |  |  | [*C. bellidifolia* subsp. *aucheri*] |
|  | = *C. hygrophila* Rupr. * |  |  |  |  |
| 6 | ***C. radchensis* Kharadze !*** | *C. radchensis* | *C. radchensis* | *C. radchensis* | [*C. bellidifolia* subsp. *aucheri*] |
| 7 | ***C. armazica* Kharadze !*** | *C. armazica* | *C. armazica* | [*C. saxifraga* subsp. *aucheri*] | [*C. bellidifolia* subsp. *aucheri*] |
| 8 | ***C. alpigena* K. Koch !*** | *C. alpigena* | *C. alpigena* | [*C. saxifraga* subsp. *aucheri*] | [*C. bellidifolia* subsp. *aucheri*] |
|  | = *C. fallax* Rupr. * |  |  |  | [*C. bellidifolia* subsp. *aucheri*] |
|  |  | **(4) Ser. *Saxifragiformes* Fed.** | **(4) Ser. *Saxifragiformes* Fed.** |  |  |
| 9 | ***C. saxifraga* M. Bieb. !*** | *C. saxifraga* | *C. saxifraga* | *C. saxifraga* subsp. *saxifraga* | *C. bellidifolia* subsp. *saxifraga* |
| 10 | ***C. ruprechtii* Boiss. !*** | *C. ruprechtii* | *C. ruprechtii* | [*C. saxifraga* subsp. *aucheri*] | [*C. bellidifolia* subsp. *aucheri*] |
|  |  | **(5) Ser. *Argunenses* Fed.** | **(5) Ser. *Argunenses* Fed.** |  |  |
| 11 | ***C. argunensis* Rupr. !*** | *C. argunensis* | *C. argunensis* | *C. saxifraga* subsp. *argunensis* | *C. bellidifolia* subsp. *argunensis* |
|  | = *C. doluchanovii* Kharadze !* | *C. doluchanovii* | *C. doluchanovii* | [*C. saxifraga* subsp. *argunensis*] | [*C. bellidifolia* subsp. *argunensis*] |
|  | = *C. meyeriana* Rupr. !* | *C. meyeriana* | *C. meyeriana* | *C. saxifraga* subsp. *meyeriana* | *C. bellidifolia* subsp. *meyeriana* |
|  |  | **(6) Ser. *Bellidifoliae* Fed.** | **(6) Ser. *Bellidifoliae* Fed.** |  |  |
| 12 | ***C. bellidifolia* Adams !*** | *C. bellidifolia* | *C. bellidifolia* | *C. bellidifolia* | *C. bellidifolia* subsp. *bellidifolia* |
|  | = *C. adami* M. Bieb. * | [*C. bellidifolia*] |  | [*C. bellidifolia*] | [*C. bellidifolia* subsp. *bellidifolia*] |
|  | = *C. sosnowskyi* Kharadze !* | *C. sosnowskyi* | *C. sosnowskyi* | [*C. bellidifolia*] | [*C. bellidifolia* subsp. *bellidifolia*] |
|  | = *C. fominii* Grossh. !* | *C. fominii* | *C. fominii* | [*C. saxifraga* subsp. *meyeriana*] | [*C. bellidifolia* subsp. *meyeriana*] |
| 13 | ***C. czerepanovii* Fed.** |  | *C. czerepanovii* |  |  |
|  |  | **(7) Ser. *Anomalae* Fed.** | **(7) Ser. *Anomalae* Fed.** |  |  |
|  | = *C. anomala* Fomin !* | *C. anomala* | *C. anomala* | [*C. circassica*] | [*C. bellidifolia* subsp. *saxifraga*] |
|  | = *C. circassica* Fomin !* | *C. circassica* | *C. circassica* | *C. circassica* | [*C. bellidifolia* subsp. *saxifraga*] |
|  |  | **(8) Ser. *Ardonenses* Fed.** | **(8) Ser. *Ardonenses* Fed.** |  |  |
|  | = *C. ardonensis* Rupr. !* | *C. ardonensis* | *C. ardonensis* | *C. ardonensis* | [*C. bellidifolia* subsp. *bellidifolia*] |
|  | = *C. kryophila* Rupr. !* | *C. kryophila* | *C. kryophila* | *C. kryophila* | [*C. bellidifolia* subsp. *bellidifolia*] |
|  |  | **(9) Ser. *Besenginicae* Fed.** | **(9) Ser. *Besenginicae* Fed.** |  |  |
| 14 | ***C. besenginica* Fomin !*** | *C. besenginica* | *C. besenginica* | *C. besenginica* | *C. bellidifolia* subsp. *besenginica* |

**Table S1.** Continued.

| **Taxon names** | | **Fedorov (****1957)** | **Kharadze (1976)** | **Oganesian (2000)** | **Victorov (2002)** |
| --- | --- | --- | --- | --- | --- |
|  |  | ***Campanula* subsect. *Scapiflorae*** | ***Campanula* sect. *Scapiflorae*** | ***Campanula* subg. *Scapiflorae*** | ***Campanula* subg. *Scapiflorae*** |
|  | = *C. pallasiana* Roem. & Schult. | [*C. dasyantha*] |  | [*C. dasyantha*] | [*C. dasyantha*] |
|  |  | **(10) Ser. *Dasyanthae* Fed.** |  |  |  |
| 15 | ***C. dasyantha* M. Bieb. !** | *C. dasyantha* |  | *C. dasyantha* | *C. dasyantha* subsp. *dasyantha* |
|  | = *C. pilosa* Pall. ex Roem. & Schult. | [*C. dasyantha*] |  | [*C. dasyantha*] | [*C. dasyantha*] |
|  | = *C. altaica* A. DC. non Ledeb. | [*C. dasyantha*] |  | [*C. dasyantha*] | [*C. dasyantha*] |
| 16 | ***C. chamissonis* Fed. ex Toyok. & Nosaka !** | *C. chamissonis* |  | *C. chamissonis* | *C. dasyantha* subsp. *chamissonis* |
|  | = *C. aldanensis* Fed. & Karav. | *C. aldanensis* |  | *C. aldanensis* | [*C. dasyantha*] |
|  |  | **(11)** **Ser. *Ledebourianae* Fed.** | **(10) Ser. *Ledebourianae* Fed.** |  |  |
| 17 | ***C. ledebouriana* Trautv. !** | *C. ledebouriana* | *C. ledebouriana* | *C. ledebouriana* |  |
| 18 | ***C. petrophila* Rupr. !** |  |  | *C. petrophila* | *C. petrophila* |
|  | = *C. zeyensi*s Amirkh. & Tavasiev * |  |  | *C. zeyensi*s | [*C. bellidifolia* subsp. *bellidifolia*] |
|  | = *C. kadargavanica* Amirkh. & Komzha !* |  |  | *C. kadargavanica* | [*C. bellidifolia* subsp. *bellidifolia*] |
|  | = *C. songutica* Amirkh. * |  |  | *C. songutica* | [*C. bellidifolia* subsp. *bellidifolia*] |
| 19 | ***C. bornmuelleri* Nábělek !** |  |  | *C. bornmuelleri* |  |
| 20 | ***C. pulvinaris* Hausskn. & Bornm. !** |  |  | *C. pulvinaris* |  |
| 21 | ***C. andina* Rupr. !** |  |  |  | *C. andina* |
|  | = *C. gumbetica* Boiss. |  |  |  | [*C. andina*] |
|  |  |  |  |  | **(4) Sect. *Saxicolae* (Boiss.) Kharadze** |
| 22 | ***C. lehmanniana*Bunge** |  |  |  | *C. lehmanniana* subsp. *lehmanniana* |
| 23 | ***C. capusii* (Franch.) Fed.** |  |  |  | *C. lehmanniana* subsp. *capusii* |
|  |  |  |  |  | **(5) Sect. *Dasystigma* (Fed.) Victorov** |
| 24 | ***C. alpina* Jacq. !** |  |  | *C. alpina* subsp. *alpina* | *C. alpina* |
| 25 | ***C.* *orbelica*** **Pančić** |  |  | *C. alpina* subsp. *orbelica* |  |
|  |  |  |  |  | **(6) Sect. *Hypopolion* (Fed.) Ogan.** |
| 26 | ***C. hypopolia* Trautv. !** |  |  |  | *C. hypopolia* |
|  |  |  |  |  | **(7) Sect. *Theodorovia* (Kolak.) Victorov** |
| 27 | ***C. minsteriana* Grossh. !** | *C. minsteriana* | *C. minsteriana* |  | *C. minsteriana* |
|  | = *C. karakuschensis* Grossh. |  |  |  | [*C. minsteriana*] |
| **Species** | | **26** | **24** | **20** | **11** |
| Subspecies | | 0 | 0 | **5** | **7** |
| Synonyms | | **6** | 0 | **15** | **28** |
