# Supplementary material for: ﻿The polyphyletic Caucasus-centred Campanula subg. Scapiflorae (Campanulaceae) revisited with a newly circumscribed C. sect. Tridentatae for its core clade
Source: PhytoKeys. 2024 Jun 25;243:149–84. doi: 10.3897/phytokeys.243.120908 (PMC11220400; doi:10.3897/phytokeys.243.120908)
Supplement: Supplementary material 4 — Primers are used for amplification and sequencing reactions [file phytokeys-243-149_article-120908__-s004.docx]

Table S2. Primers are used for amplification and sequencing reactions. M13F and M13R were used for sequencing, all other primers were used for amplification.

| **Primers** | **Sequences** | **Authors** |
| --- | --- | --- |
| PlpetB1365F | TTGACYCGTTTTTATAGTTTAC | Löhne and Borsch, 2005 |
| CApetD-324R | ATCCCYTGTTTCACTCCGATAG | designed by Schäferhoff, in this study |
| CApetD-194F | CAGGCTCCGTAARATCCAG | designed by Schäferhoff, in this study |
| PlpetD738R | AATTTAGCYCTTAATACAGG | Löhne and Borsch, 2005 |
| rpl16F | CTATGCTTAGTGTGTGACTC | Löhne and Borsch, 2007 |
| CAMrpl16-690R | CGAATAGATGAATTGGTCTCTGG | In this study |
| CAMrpl16-640F | GGATTCATTGATCTGGATGG | In this study |
| rpl16R | TCTTCCTCTATGTTGTTTACG | Campagna and Downie,1998 |
| trnK-F | GGGTTGCTAACTCAATGGTAGAG | Wicke and Quandt, 2009 |
| CAMtrnK-570R | CCTCGGATACAAGTAAAC | In this study |
| CAMtrnK-400F | CTTCATTCTACAATGGAGAGG | In this study |
| CAMmatK-170R | CGAGGAATTAACCKTTTCAC | In this study |
| CAMtrnK-880F | GCTCGATAGATTTCAACAAC | In this study |
| CAMmatK720R | ATGGATTCGTATTCACATAC | Jones et al., 2017 |
| CAMmatK507F | TTCAAGCTCTTCGCTACTGG | Jones et al., 2017 |
| CAMmatK530F | AAAGATGCTTCGTCTTTGC | Jones et al., 2017 |
| CAMmatK-1020R | GTACCGTTGAAGTTCTTC | In this study |
| CAMmatK-890F | GTCTCTTGTGATGAATAAATGG | In this study |
| CAMmatK-1400R | GAAGAAGCTCTGGGAAAGG | In this study |
| CAMmatK-1300F | CTTCAACTTTCTTGTGCTAG | In this study |
| trnK-2R | AACTAGTCGGATGGAGTAG | Johnson & Soltis, 1995 |
| psbA5'R | AACCATCCAATGTAAAGACGGTTT | Shaw et al., 2005 |
| M13F | GTAAAACGACGGCCAGT | Messing, 1983 |
| M13R | GCGGATAACAATTTCACACAGG | Messing, 1983 |
